# Roles of Self‐Stigma and Meaning in the Relationship Between Age and Loneliness: A Perspective From Gerotranscendence

**DOI:** 10.1002/pchj.70115

**Published:** 2026-07-26

**Authors:** Han Xiao, Melina Malli, Pamela Qualter, Manuela Barreto, Christina Victor, Theodore D. Cosco, Guangyu Zhou

**Affiliations:** ^1^ School of Psychological and Cognitive Sciences, Peking University Beijing China; ^2^ Oxford Institute of Population Ageing University of Oxford Oxford UK; ^3^ Beijing Key Laboratory of Behavior and Mental Health Peking University Beijing China; ^4^ Key Laboratory of Machine Perception, Ministry of Education Beijing China; ^5^ Manchester Institute of Education University of Manchester Manchester UK; ^6^ Psychology Department University of Exeter Exeter UK; ^7^ College of Health and Life Sciences Brunel University London UK; ^8^ Department of Gerontology Simon Fraser University Vancouver Canada

**Keywords:** age, gerotranscendence, loneliness, loneliness self‐stigma, meaning of loneliness

## Abstract

Drawing on the theory of gerotranscendence, this research aimed to gain a more comprehensive and nuanced understanding of the age‐loneliness relationship and underlying factors. Data from the BBC Loneliness Experiment (*n* = 6875, 67.80% female, *M*_age_ = 49.26, Range_age_ 16–94) were analyzed using polynomial regression and Multi‐Group Structural Equation Modeling (MG‐SEM) for the quantitative data, and complemented by Latent Dirichlet Allocation (LDA) for free‐text data. The results showed: (1) there was a non‐linear, inverted *U*‐shaped relationship between age and loneliness, with an inflection point at 39.2 years of age; (2) in the older age group, loneliness self‐stigma and the meaning of loneliness played parallel mediating roles between age and loneliness, whereas no mediating effect was observed in the younger age group; (3) these mediating effects primarily attenuated the loneliness frequency and loneliness intensity rather than loneliness duration; (4) two overarching topics of loneliness (i.e., *Limited Social Functions* and *Unsatisfactory Social Relationships*) were identified, with differences between the two age groups. This study further enriches the understanding of gerotranscendence in the loneliness domain and provides suggestions for tailoring precision interventions (i.e., self‐stigma, meaning‐making) to distinct life stages and specific loneliness dimensions.

## 1 Introduction

Loneliness may occur when subjective feelings of pain or unhappiness are caused by the discrepancy between an individual's actual social relationships and ideal social relationships (de Jong‐Gierveld et al. 2018; Lim et al. 2023). Nowadays, loneliness is becoming an important public health issue (Badcock et al. 2022; Lim et al. 2020), which is seriously damaging people's social functions and leading to cognitive impairment (Huang et al. 2023; Tan et al. 2020). Although loneliness can emerge at any age (Mund et al. 2020), existing literature regarding the relationship between age and loneliness remains notably inconsistent. Some studies propose a linear decline in loneliness as individuals age (Suanet et al. 2024); others report evidence of a linear increase (Hülür et al. 2016), and several suggest complex non‐linear patterns (Hawkley et al. 2022; Victor and Yang 2012). Accordingly, it is warranted to examine the precise age‐loneliness relationship pattern and to identify the inflection age point (if any) in this relationship within a large sample. Crucially, while statistical trends have been widely debated, the underlying psychological mechanisms and nuanced differences in this relationship should be further elucidated.

The theory of gerotranscendence conceptualizes aging as a process of development and a transformation in meta‐perspective (Tornstam 1997), which includes three dimensions: the *cosmic* (a sense of connection with existence and the universe rather than a sense of isolation), the *self* (a decrease in self‐centeredness and enhanced self‐integration), and *social and personal relationships* (a reduced need for superficial interactions and increased need for deep bonds). In the context of loneliness, one potential consequence of these dimensions is their influence on how older adults cognitively cope with loneliness: reducing loneliness self‐stigma and enhancing meaning‐making. For instance, older adults may adopt a perspective that normalizes loneliness as a universal experience, thereby reducing self‐focus and loneliness self‐stigma (i.e., the *cosmic* and the *self* dimensions) (Tornstam 2011). Simultaneously, the shift in the need for social relationships and self‐integration (i.e., the *self* and the *social and personal relationships* dimensions) enables older adults to reappraise loneliness not as a deficit, but as a meaningful space for freedom and existential clarity (Baltes and Dickson 2001; Morlett Paredes et al. 2021).

Therefore, the current study aims to determine whether a non‐linear age‐loneliness relationship exists, identify its precise inflection point, and uncover the psychological mechanisms (i.e., self‐stigma and meaning) underlying the association between age and loneliness.

### 1.1 Loneliness Self‐Stigma

Self‐stigma is the incorporation of a negative stereotype into one's identity (Bos et al. 2013; Criswell 2025). Loneliness is often stigmatized, with lonely individuals being attributed more negative traits—such as being less adjusted, less competent, and less likable—compared to those who are not lonely (Department for Culture, Media and Sport 2023; Kerr and Stanley 2021). Research indicates that stigma can intensify feelings of loneliness, creating barriers to forming social connections (Pitman et al. 2018). However, gerotranscendence suggests that older adults demonstrate a decrease in self‐centeredness and ego‐preoccupation, coupled with greater self‐integration (Tornstam 2011). Specifically, older adults are less susceptible to external judgments and more attuned to their internal feelings, resulting in diminished feelings of shame or embarrassment when experiencing loneliness. Research demonstrates that disengaging from negative social evaluations (e.g., feeling shame and embarrassment about loneliness) and facilitating authentic emotional expression in later life are highly beneficial for health and well‐being (Milligan et al. 2016). We propose that this age‐related cognitive change elucidates why older adults internalize the stigma of loneliness to a lesser degree than younger adults, buffering them against loneliness.

Additionally, the widespread belief that loneliness is particularly prevalent among older adults (Gardiner et al. 2020; Rapolienė and Aartsen 2022) may normalize the experience for this age group, reducing its stigmatizing impact. Conversely, younger adults are usually framed as highly social, rendering loneliness less normative and potentially more stigmatizing for them (Malli et al. 2023; Pikhartova et al. 2016). Driven by elevated loneliness self‐stigma, people often actively conceal their condition to avoid negative social judgments (Mental Health Foundation 2022), which is a maladaptive coping strategy that impedes help‐seeking and ultimately aggravates their experience of loneliness (Kantar Public 2016; Ko et al. 2022).

Taken together, these ideas lead us to expect that loneliness self‐stigma plays a mediating role in the relationship between age and loneliness.

### 1.2 Meaning of Loneliness

Meaning‐making involves construing an experience as comprehensible and significant (Martela and Steger 2016; Park 2010). Existing research and the wider media overwhelmingly portray the meaning of loneliness as negative (Ferguson 2018; Prohaska et al. 2020). However, emerging empirical evidence challenges this uniformly negative stereotype by revealing that loneliness can also be defined variously (Switsers et al. 2023; Victor et al. 2019): when regarded as the painful experience of loss, exclusion and sadness, it is appraised as negative; when regarded as enabling personal creativity, growth, and freedom, it is appraised as positive (de Jong‐Gierveld et al. 2018; Wotherspoon 2024).

Establishing new connections and maintaining social relationships are important to younger adults (Maes et al. 2019). Conversely, the narrowing of social connections (e.g., due to retirement, bereavement, and illness) is seen as an inevitable part of growing older (Cohen‐Mansfield and Eisner 2020; Vedder et al. 2022). From a gerotranscendent perspective (Tornstam 1997, 2011), older adults exhibit a greater capacity to acknowledge and accept loneliness, cognitively appraising and conceptualizing it as a meaningful experience that is inherent to the purpose of life (Mansfield et al. 2021).

The selective optimization with compensation theory (Baltes and Baltes 1990) also suggests that people may employ selective optimization to cope with the losses associated with aging. In response to perceived threats, such as shrinking social networks, older adults might seek to alleviate lonely feelings through two strategies of meaning‐making: reaffirming meaning in areas where life is threatened (e.g., reflecting on the meaning of loneliness) and redirecting their attention to other aspects (e.g., finding positive meaning in loneliness). The latter strategy, known as compensation (Baltes and Dickson 2001; Morlett Paredes et al. 2021), helps older adults protect the comprehensibility and significance of their existence, while also reducing loneliness (Borawski et al. 2022).

Therefore, this study hypothesizes that the meaning of loneliness (i.e., how positively it is appraised) functions as a mediating factor between age and loneliness.

### 1.3 Loneliness Dimensions

In addition to understanding the effects of the factors mentioned above, it is necessary to comprehensively examine and carefully distinguish the different dimensions of loneliness (Weiss 1975). Loneliness should be viewed from the same perspective as other emotion‐focused experiences (such as depression) with frequency (e.g., how often loneliness happens: never, very often), intensity (e.g., what is the level of distress the loneliness causes: severe, moderate, mild), and duration (e.g., how long loneliness lasts: a day, a week, a month, a year) (Qualter et al. 2021).

Therefore, one auxiliary aim of the current study is to explore if and how these mechanisms differ across the loneliness frequency, intensity, and duration.

### 1.4 Textual Semantic Topics of Loneliness

Moreover, to deepen the interpretation of the quantitative findings, another auxiliary aim of the current study is to explore the textual semantic topics, which further capture the nuanced phenomenology of the loneliness experience within the framework of gerotranscendence. This study incorporated Latent Dirichlet Allocation (LDA) topic modeling. LDA is a statistical model that leverages an unsupervised machine learning approach to discover the underlying semantic structure in textual data (Blei et al. 2003; Hagg et al. 2022). The latent semantic structure consists of a set of related topics identified by co‐occurring words, rather than relying on predefined topical assumptions.

In the context of the current study, textual semantic analysis could facilitate the understanding of age‐related differences in loneliness, enriching our investigation into the loneliness self‐stigma, the meaning of loneliness, and the dimensions of loneliness.

### 1.5 Overview of the Current Study

Collectively, by integrating the theoretical frameworks of gerotranscendence, stigmatization, and meaning‐making, this study aims to investigate:

Hypothesis 1 *Age exhibits a non‐linear relationship with loneliness, characterized by a precise inflection point*.

Hypothesis 2 *The loneliness self‐stigma plays a mediating role in the relationship between age and loneliness*.

Hypothesis 3 *The meaning of loneliness plays a mediating role in the relationship between age and loneliness*.

Hypothesis 4 (exploratory) *The magnitude of the mediating effects (i.e., loneliness self‐stigma and meaning of loneliness) varies across the three loneliness dimensions (i.e., frequency, intensity, and duration)* **(H4a)**. When the sample is divided at the identified inflection point, the characteristics of textual semantic topics relating to loneliness are expected to differ between age groups.

**FIGURE 1:**
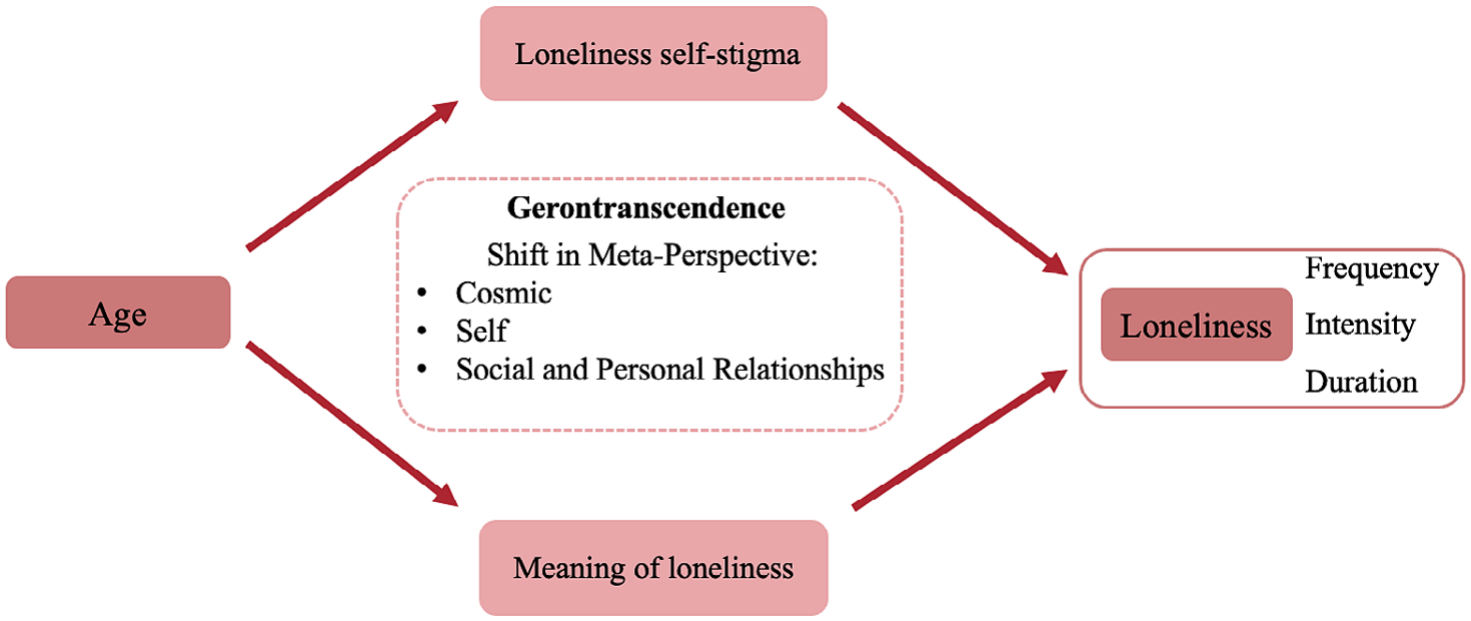
Conceptual framework of the current study. Dashed boxes denote the foundational theoretical constructs derived from theory of gerotranscendence (Tornstam 1997, 2011). Solid boxes denote the key variables operationalized within the context of the current study (i.e., age, loneliness self‐stigma, meaning of loneliness, loneliness frequency, loneliness intensity, and loneliness duration).

The conceptual framework of the current study is shown in Figure 1.

## 2 Methods

### 2.1 Participants

Participants were from a large‐scale global study—the British Broadcasting Corporation (BBC) Loneliness Experiment. The data were collected between February and May 2018. The survey received ethical approval from the University of Manchester (Reference No. 2017‐2710‐4594). The ethical guidelines of the British Psychological Society and the 2013 Declaration of Helsinki were followed for this survey. The online survey was launched through BBC Radio 4 and the BBC World Service and covered elsewhere on radio, TV, and social media, extending the coverage of the study.

An a priori power analysis was conducted using a structural equation model (SEM) sample size calculator (Soper 2025) to determine the sample size needed for testing the parallel mediation model. To detect a medium‐sized mediating effect with a desired power of 0.80 and a significance level of 0.05 (Cohen 1988; Westland 2010), a minimum of 2900 participants was required. In the current study, we analyzed data from 6875 participants (16–94 years old, *M* = 49.26, SD = 15.38; 32.20% male; 71.60% residing in the UK) who provided data for the variables of interest in this study from the BBC Loneliness Experiment. The detailed characteristics of participants are shown in Supporting Information S1. Participants took approximately 40 min to complete the survey. Information was collected about age, loneliness (including loneliness frequency, intensity, and duration), loneliness self‐stigma, and the meaning of loneliness.

### 2.2 Measures

#### 2.2.1 Loneliness

Loneliness was measured using the 4‐item UCLA Loneliness Scale (Russell et al. 1980): “Do you feel a lack of companionship?” “Do you feel left out?” “Do you feel isolated from others?” *and* “Do you feel in tune with people around you?” (reverse‐coded). We adapted the response options by asking about frequency (“How often does that happen?”, from 1 = *never* to 5 = *very often*), intensity (“How intense is that feeling?”, from 1 = *not at all* to 5 = *very intense*), and duration (“How long does that feeling last when it occurs?”, 1 = *hours*, 2 = *days*, 3 = *weeks*, 4 = *months*, 5 = *longer*) of loneliness (Qualter et al. 2021). Each subscale was averaged across the four items to obtain total scores for these three loneliness dimensions. With higher scores indicated greater loneliness. Qualter et al. (2021) obtained reliable Cronbach's *α* for frequency, intensity, and duration = 0.85, 0.86, and 0.88 respectively. In this study, the measures were reliable: Cronbach's *α* for frequency, intensity, and duration = 0.83, 0.82, and 0.86, respectively (and for all items = 0.95). For more details about justifying the decomposition of loneliness into three dimensions (frequency, intensity, and duration) in the current sample, please refer to Supporting Information S2.

Additionally, participants answered a free‐text question to express their attitudes and experiences of loneliness on a voluntary basis, and no word limit was imposed on the length of their response. Among the 6875 participants in this study, 5682 of them (32.20% male) answered the question. The MANOVA showed that between the participants who answered the questions and those who did not, there were no significant differences in terms of loneliness (including loneliness frequency, intensity, and duration; *Fs*_(1, 6874)_ ranged from 0.11 to 2.70, *ps* > 0.10, partial *η* ^2^ < 0.001); there were no significant differences in terms of SES‐related indicators (including subjective social status, years of education, and employment status; *Fs*_(1, 6874)_ ranged from 0.52 to 0.55, *ps* > 0.46, partial *η* ^2^ < 0.001). A detailed description of SES‐related indicators is provided in the *Covariates* section.

#### 2.2.2 Loneliness Self‐Stigma

Loneliness self‐stigma was measured with three items: “When I feel lonely, I feel ashamed about it,” “When I feel lonely, I am too embarrassed to admit it to others”, *and* “When I feel lonely, I don't talk to others about it.” The measurement obtained a reliable Cronbach's *α* = 0.80 (Barreto et al. 2022). Participants responded to each item on a 7‐point Likert‐type scale (from 1 = *strongly disagree* to 7 = *strongly agree*). Item‐level responses for these scales served as the observable variable and were synthesized to create a continuous composite index. Higher scores are indicative of greater loneliness self‐stigma. In this study, the measure was reliable (Cronbach's *α* = 0.80).

#### 2.2.3 Meaning of Loneliness

We ascertained the meaning of loneliness using a multiple‐choice question, “What does loneliness mean to you?”, which was developed specifically for the BBC Loneliness Experiment. Participants were required to choose as many items as they thought applied. It contained 10 items, including 3 positive items (e.g., loneliness means “Spending time focusing on yourself”, “Being on your own”) and 7 negative items (e.g., loneliness means “Sadness”, “Feeling vulnerable”). The answer for each item was coded as 0 = *not selected* and 1 = *selected*. To capture the overall valence of meaning ascribed to loneliness, negative items were reverse‐coded, and a sum score was calculated across all items. Higher scores indicate a greater degree of positive meaning of loneliness. This aggregative scoring approach for mixed‐valence checklists aligns with the similar practices in psychometric research (Nunnally and Bernstein 1994; Yesavage et al. 1982; Zuckerman et al. 1983). The confirmatory factor analysis yielded the following fit indices: *χ* ^2^/df = 26.26, CFI = 0.90, TLI = 0.83, SRMR = 0.07, and RMSEA = 0.06.

#### 2.2.4 Covariates

According to the previous evidence, gender (dummy: 0 = *female*, and 1 = *male*), socioeconomic status (SES), solitude (actively seeking and enjoying being alone), and living circumstances (whether living alone) are associated with loneliness (Switsers et al. 2023; Weinstein et al. 2023); thus, the current study included these factors as covariates.

Subjective social status, income, education, and employment are important indices of SES (Ganzeboom et al. 1992; Hoebel and Lampert 2020). Participants were asked to indicate their status within their country using the 10‐rung ladder (MacArthur Subjective Social Status measure, MSSS) (Adler et al. 2000). The subjective evaluation of how well their financial needs were met by their income was measured using a 3‐point Likert‐scale (1 = *poorly*, 2 = *fairly well*, 3 = *very well*) (Pfeiffer 1975). Education was measured in years (continuous), and employment status was dichotomized as inactive (coded as 0 = *not active in the labor market*, including retired, student, and unemployed) or active (coded as 1 = *active in the labor market*, including full‐time work and part‐time work) (Pan et al. 2023). The above indices were standardized separately and summed to generate an overall indicator of participants' SES (continuous).

Solitude was measured using 6 items on a 3‐point Likert‐type scale (e.g., “To think something over without distraction, I want to be alone”), and scores were summed after reversing the scoring of the reverse‐scored items. Higher scores are indicative of greater solitude (Barreto et al. 2021). To our knowledge, no prior studies have reported on the psychometric properties of the solitude scale. In the current study, the Cronbach's *α* of the solitude scale was 0.72. Living circumstances were dichotomized by the question “Do you live alone?” (0 = *no*, 1 = *yes*).

### 2.3 Data Analysis

The overall rate of missing data in the current sample was 0.70%. The full‐information maximum likelihood (FIML) approach was used to handle missing data robustly, which could mitigate estimation bias stemming from nonnormal distribution and is highly effective for datasets with missing data well below the 30% threshold (Graham 2009). Statistical analyses were conducted using IBM SPSS Statistics (version 29) and RStudio (version 2024.4.2.764) for quantitative data, and in Python (version 3.11.4) for textual data. The primary analytical codes of the current study are available on OSF (https://osf.io/ftxc2/overview).

#### 2.3.1 Non‐Linear Relationship and Inflection Point Test

First, a preliminary descriptive analysis was conducted to calculate sums and/or means, standard deviations, and correlations among the variables. This enabled us to characterize the loneliness experience of our study population. Then, polynomial regression modeling (comparing linear *age*, quadratic *age* ^*2*^, and cubic *age* ^*3*^ functional forms) was employed to explicitly test for non‐linear patterns in the relationship between age and loneliness and to identify the inflection age point in this relationship.

#### 2.3.2 Structural Equation Modeling

To further test the gerotranscendence‐based hypotheses, the sample was subsequently stratified into different life‐stage groups based on the inflection age point. Multi‐Group Structural Equation Modeling (MG‐SEM) was utilized to evaluate the parallel mediating roles of loneliness self‐stigma and meaning of loneliness between age and loneliness frequency, intensity, and duration across the groups. To test whether the robustness of the parameter estimates was maintained across groups, measurement invariance was examined before MG‐SEM. Model specification and estimation path analyses were conducted using the lavaan package in RStudio.

In the model specification, age, the dimensions of loneliness, meaning of loneliness, and the covariates were entered as observed variables, and the loneliness self‐stigma was entered as a latent variable measured by 3 items (i.e., ashamed, embarrassed, and do not talk). Considering the scaling disparities among variables with different original metrics (e.g., chronological age in years, binary dummy codes, and aggregated Likert means), the variables were transformed into standardized *Z*‐scores before estimation (Kline 2023). To conservatively account for any potential deviations from multivariate normality, all models were estimated using the Maximum Likelihood Robust Estimator (MLR) (Rhemtulla et al. 2012). To generate bias‐corrected confidence intervals (MacKinnon 2008), the bootstrap samples were set as 5000. Where the 95% confidence intervals (CI) did not include zero, it indicated that the mediating effect was significant (Preacher and Hayes 2008). Additionally, cutoff values for fit indices were employed, including the *χ* ^2^/df ratio, the Tucker‐Lewis index (TLI), the comparative fit index (CFI), the root mean square error of approximation (RMSEA), and standardized root mean square residual (SRMR). To prevent biased estimates due to outcome redundancy, the residual covariances among loneliness frequency, intensity, and duration were freely estimated across all structural models. Moreover, to examine the relative magitude of the mediation across the three loneliness dimensions, the absolute values of mediating effects for loneliness frequency, intensity, and duration were compared (Cheung 2007; Liu et al. 2024). To evaluate between‐group differences in the mediating effects, the custom contrasts were specified to calculate the exact effect size differences between groups with a 5000‐resample bootstrapping procedure (see Supporting Information S3). Owing to the large sample size, a more rigorous criterion of *p* < 0.01 (rather than *p* < 0.05) was utilized. Interpretations emphasized standardized effect sizes to ensure that the reported effects are not only statistically significant but also theoretically and practically meaningful.

Finally, to ensure the robustness of the findings against the demographic characteristics of the current sample, four additional analyses were conducted. First, an additional 2‐group MG‐SEM was tested, controlling for macro‐level cultural variance by assigning each participant a score on Hofstede's Individualism Index (IDV, 0–100 scale; Hofstede 1997) based on their country of residence. Second, an additional 4‐group MG‐SEM (stratified by both inflection point and gender) was tested for gender invariance. Third, to validate the results from a standard lifespan‐developmental perspective, an additional MG‐SEM was conducted to test the robustness of the primary findings in theoretically defined age cohorts (i.e., emerging adulthood, early‐middle adulthood, later‐middle adulthood, and later adulthood) (Arnett 2000; Morbey et al. 2025; Srivastava et al. 2003). Fourth, the competing sequential (chain) mediation models were evaluated and compared with the primary parallel mediation models. Full details of additional analyses are available in Supporting Information S4.

#### 2.3.3 Latent Dirichlet Allocation

Gensim and NLTK libraries were mainly used for textual semantic analysis. Participants who failed to provide valid open‐ended responses (coded as −99) were excluded from the corpus. The raw text corpus (total word count = 155,343) underwent a series of preprocessing steps to facilitate subsequent computational analysis. Preprocessing is a natural‐language‐processing (NLP) workflow to clean and structure raw text, making it suitable for analysis techniques like Latent Dirichlet Allocation (LDA) topic modeling (Maier et al. 2021). The preprocessing pipeline was implemented using Natural Language Toolkit package (NLTK) (Bird et al. 2009). First, following the identification of the inflection age point, the textual dataset was partitioned. Second, the text was then tokenized into individual words, with corrections made for spelling and spacing. Third, the remaining words underwent lemmatization, a process that standardizes different versions of a word to its base form, thereby improving the coherence and accuracy of the resulting topic model. Fourth, to ensure the analysis focused on words with substantive meaning, hyperlinks, special characters, articles, conjunctions, colloquial language (e.g., “kinda,” “yeah”), and non‐lexical strings (e.g., characters composed only of consonants or vowels) were removed using a custom and standard English stop‐word dictionary (Bird et al. 2019). Subsequently, the model was initialized with a fixed random seed (random_state = 100) and trained over 10 passes, utilizing auto alpha to optimize the topic‐word distributions.

Determining the optimal number of topics relied on both quantitative coherence metrics and qualitative theoretical evaluation, with a topic count (*k*) ranging from one to six. This selection process was guided by quantitative model indices (by Gensim), including Perplexity, Topic Coherence, and Normalized Pointwise Mutual Information (NPMI) (Hagg et al. 2022; Kapadia 2019). Specifically, Perplexity denotes the difference between the expected value according to the model and the observed value, topic coherence measures the semantic similarity of words within a topic, and NPMI assesses word co‐occurrence. An optimal model demonstrates low perplexity, high topic coherence, and high NPMI values. While the utilization of quantitative coherence metrics narrowed the candidate models, existing research in topic modeling suggests that purely quantitative metrics alone do not always produce interpretable models (Chang et al. 2009). Therefore, to ensure the interpretability and plausibility of the final model and to avoid fragmented and redundant subtopics, the selection was confirmed through a qualitative review considering the metrics and semantic overlap. Finally, the visualization of the output was produced by pyLDAvis (Mabey 2020).

## 3 Results

### 3.1 Inverted *U*‐Shaped Relationship Between Age and Loneliness

Harman's single‐factor test was used to examine common method bias (Aguirre‐Urreta and Hu 2019; Lindell and Whitney 2001). The results of the unrotated factor analysis showed that there were 9 factors with eigenvalues greater than 1. The first principal factor accounted for 24.22% (< 40%) of the variance, indicating that the common method bias was not serious in this study (Zhou and Long 2004). In addition, there was no multicollinearity among those tested variables (Tolerance > 0.20, VIF < 5) (Shrestha 2020), and further analyses were conducted. Table 1 displays the correlations for key variables. Age was negatively correlated with loneliness (*r*_frequency_ = −0.08, *r*_intensity_ = −0.11, and *r*_duration_ = −0.05, *ps* < 0.001) and loneliness self‐stigma (*rs* < −0.08, *ps* < 0.001), and positively correlated with positive meaning of loneliness (*r* = 0.28, *p* < 0.001).

**TABLE 1:** Correlations for key variables.

	*M*	SD	1	2	3	4	5	6	7	8
1. Age	49.26	15.38	—							
2. Loneliness frequency	2.83	1.09	−0.08***	—						
3. Loneliness intensity	2.85	1.07	−0.11***	0.89***	—					
4. Loneliness duration	3.10	1.39	−0.05***	0.87***	0.83***	—				
5. Stigma_ashamed	3.98	1.94	−0.21***	0.30***	0.33***	0.28***	—			
6. Stigma_embarrassed	4.71	1.88	−0.15***	0.32***	0.32***	0.29***	0.64***	—		
7. Stigma_do not talk	5.10	1.81	−0.08***	0.29***	0.27***	0.26***	0.38***	0.66***	—	
8. Meaning of loneliness	3.04	2.00	0.28***	−0.22***	−0.26***	−0.21***	−0.28***	−0.22***	−0.12***	—

*Note: N* = 6875.

*** *p* < 0.001.

**FIGURE 2:**
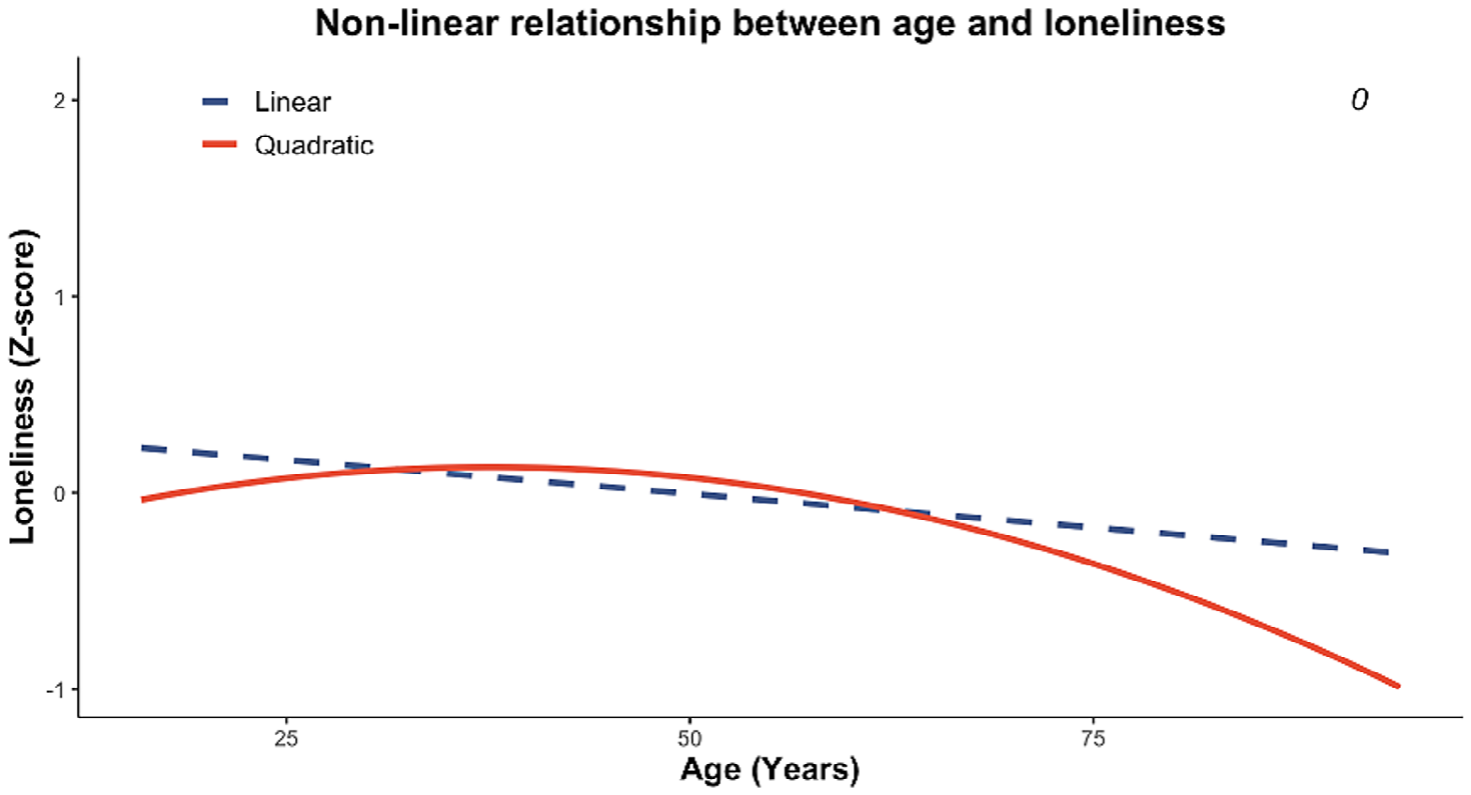
Inverted *U*‐shaped relationship between age and loneliness.

The results of the relationship test between age and loneliness are shown in Table 2. The linear relationship revealed that loneliness decreased with age. Moreover, a significant inverted *U*‐shaped relationship between age and loneliness was identified. Specifically, loneliness peaks at an inflection point at the age of 39.2, after which it begins a decline. The non‐linear inverted *U*‐shaped relationship is shown in Figure 2.

**TABLE 2:** Variance explained by linear, quadratic, and cubic models.

		β	*t*	*p*	*R* ^*2*^	*∆R* ^*2*^	*∆F*	*p* (∆)
Linear		−0.007	−9.27	< 0.001	0.18			
Non‐linear	Quadratic	−0.0004	−8.46	< 0.001	0.19	0.01	71.53	< 0.001
	Cubic	0.000	−1.60	0.110	0.20	0.0003	2.55	0.110

*Note:* ∆ refers to quadratic index subtracts linear index or cubic index subtracts quadratic index. Adjusting for gender, SES, living alone, and solitude.

### 3.2 Parallel Mediation Analysis

Based on the identified inflection point (age = 39.2), the whole sample was divided into two life‐stage groups: younger age group (<= 39.2 years, *n* = 1922) and older age group (> 39.2 years, *n* = 4953). The parallel mediation model revealed a satisfactory fit to the data in both groups (younger age group: *χ* ^2^/df = 8.81, *p* < 0.001, CFI = 0.98, TLI = 0.94, SRMR = 0.06, RMSEA = 0.03; older age group: *χ* ^2^/df = 8.81, *p* < 0.001, CFI = 0.99, TLI = 0.98, SRMR = 0.04, RMSEA = 0.01). The constrained metric invariance model showed no significant decrement in model fit compared to the configural invariance model (*∆χ* ^2^ = 1.34, *p* = 0.52) between both age groups, ensuring the constructs were operationalized consistently.

**FIGURE 3:**
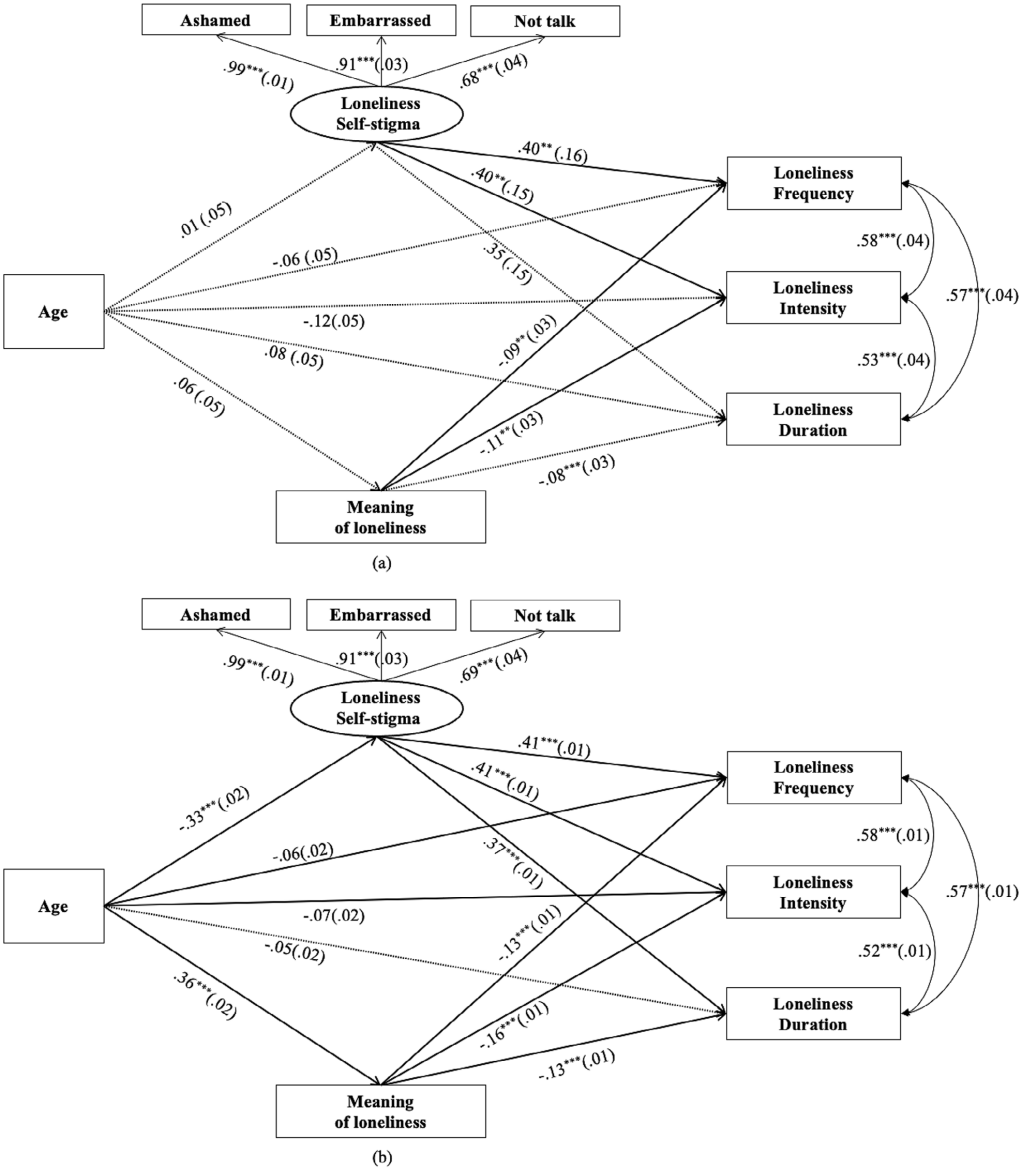
The parallel mediation models: (a) is the parallel mediation model of the younger age group, (b) is the parallel mediation model of the older age group. Adjusting for gender, SES, living alone, and solitude. The value outside the parentheses is standard regression coefficient, the value inside the parentheses is standard error. The straight line represents a significant path, and the dashed line represents an insignificant path. ***p < 0.001, **p < 0.01.

After adjusting for gender, SES, living alone, and solitude, the results of the parallel mediation effect analysis for the two groups are shown in Figure 3 and Table 3. The results indicated the age‐specificity of the mediating mechanisms: neither loneliness self‐stigma nor meaning of loneliness demonstrated parallel mediating effects in the younger age group. In contrast, the loneliness self‐stigma and meaning of loneliness exhibited significant and substantial mediating effects in the older age group. Specifically, in the younger age group, the *R* ^2^ for loneliness frequency, intensity, and duration is 0.25, 0.24, and 0.20, respectively. The parallel mediating pathways of loneliness self‐stigma and meaning of loneliness accounted for 16.67% and 17.65% of the total model effect, respectively. In the older age group, the *R* ^2^ for loneliness frequency, intensity, and duration is 0.33, 0.35, and 0.28, respectively. The parallel mediating pathways of loneliness self‐stigma and meaning of loneliness accounted for 54.48% and 21.01% of the total model effect, respectively.

**TABLE 3:** Parallel mediating effects of loneliness self‐stigma and meaning of loneliness.

Group	Pathway			*β*	SE	*p*	Boot LLCI	Boot ULCI
Younger age group	Indirect effects							
	Age →	Loneliness self‐stigma	→ Loneliness frequency	0.006	0.019	0.762	−0.032	0.043
			→ Loneliness intensity	0.006	0.019	0.761	−0.031	0.043
			→ Loneliness duration	0.005	0.017	0.762	−0.028	0.038
	Total indirect effect of loneliness self‐stigma			0.017	0.055	0.762	−0.091	0.124
	Age →	Meaning of loneliness	→ Loneliness frequency	−0.006	0.005	0.266	−0.015	0.004
			→ Loneliness intensity	−0.007	0.006	0.247	−0.019	0.005
			→ Loneliness duration	−0.005	0.005	0.268	−0.014	0.004
	Total indirect effect of meaning of loneliness			−0.018	0.016	0.256	−0.048	0.013
	Total effects of the model							
				−0.102	0.145	0.483	−0.385	0.182
Older age group	Indirect effects							
	Age →	Loneliness self‐stigma	→ Loneliness frequency	−0.134	0.008	< 0.001	−0.148	−0.119
			→ Loneliness intensity	−0.136	0.008	< 0.001	−0.151	−0.120
			→ Loneliness duration	−0.120	0.007	< 0.001	−0.134	−0.106
	Total indirect effect of loneliness self‐stigma			−0.389	0.022	< 0.001	−0.431	−0.347
	Age →	Meaning of loneliness	→ Loneliness frequency	−0.048	0.005	< 0.001	−0.058	−0.037
			→ Loneliness intensity	−0.057	0.006	< 0.001	−0.068	−0.046
			→ Loneliness duration	−0.046	0.006	< 0.001	−0.057	−0.035
	Total indirect effect of meaning of loneliness			−0.150	0.016	< 0.001	−0.181	−0.120
	Total effects of the model							
				−0.714	0.058	< 0.001	−0.828	−0.600

*Note:* Adjusting for gender, SES, living alone, and solitude.

**FIGURE 4:**
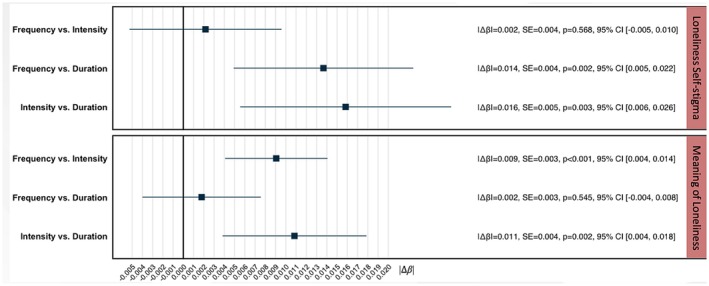
The absolute differences in the mediating effects across the dimensions of loneliness within the older age group. |∆ 𝛽| is the absolute difference in the mediating effect. Adjusting for gender, SES, living alone, and solitude.

Subsequently, the mediating effects comparison for three dimensions of loneliness (i.e., frequency, intensity, and duration) was further conducted within the older age group. The forest plot (see Figure 4) demonstrated the absolute differences in the standardized indirect effects (|∆*β*|). The mediating effect of loneliness self‐stigma in the relationship between age and loneliness duration was weaker than that in the relationship between age and loneliness frequency, and weaker than that in the relationship between age and loneliness intensity. Additionally, the mediating effect of the meaning of loneliness in the relationship between age and loneliness intensity was stronger than that in the relationship between age and loneliness frequency, and stronger than that in the relationship between age and loneliness duration.

Moreover, the contrast analyses indicated that the mediating effects were significantly stronger in the older age group compared to the younger age group. The results for all six contrast tests (i.e., the group differences for the mediating effects of loneliness self‐stigma between age and loneliness frequency, intensity, and duration, separately; the group differences for the mediating effects of meaning of loneliness between age and loneliness frequency, intensity, and duration, separately) are shown in Supporting Information S3.

### 3.3 Robustness and Sensitivity Analyses

Additional analyses indicated the robustness of the primary findings across demographic variations (i.e., cultural individualism, gender, and developmental stage) and competing sequential mediation models. First, after introducing IDV as a covariate into the model, structural paths and indirect effects remained consistent with the model without adjusting the IDV. Second, when the sample was stratified into a 4‐group MG‐SEM to account for gender (Younger Males, Younger Females, Older Males, and Older Females), the magnitude and direction of the mediational pathways remained consistent, indicating no significant gender differences in the underlying gerotranscendent mechanisms. Third, consistent with the primary findings, the older groups (i.e., later‐middle adulthood and later adulthood) demonstrated parallel mediating effects, whereas the younger groups (i.e., emerging adulthood and early‐middle adulthood) exhibited no significant mediating effects through the loneliness self‐stigma or the meaning of loneliness. These findings conceptually corroborated the data‐driven inflection point in the primary findings and aligned with the theory of gerotranscendence, indicating that the capacity to derive positive meaning from loneliness and experience lower self‐stigma to buffer loneliness is a distinct developmental transformation to later life stages. Fourth, the sequential mediating models failed to reach the meaningful magnitudes of the indirect effects and to meet the generally accepted cutoff criteria for adequate model fit. The results of the additional analyses are shown in Supporting Information S4.

### 3.4 Latent Dirichlet Allocation

**FIGURE 5:**
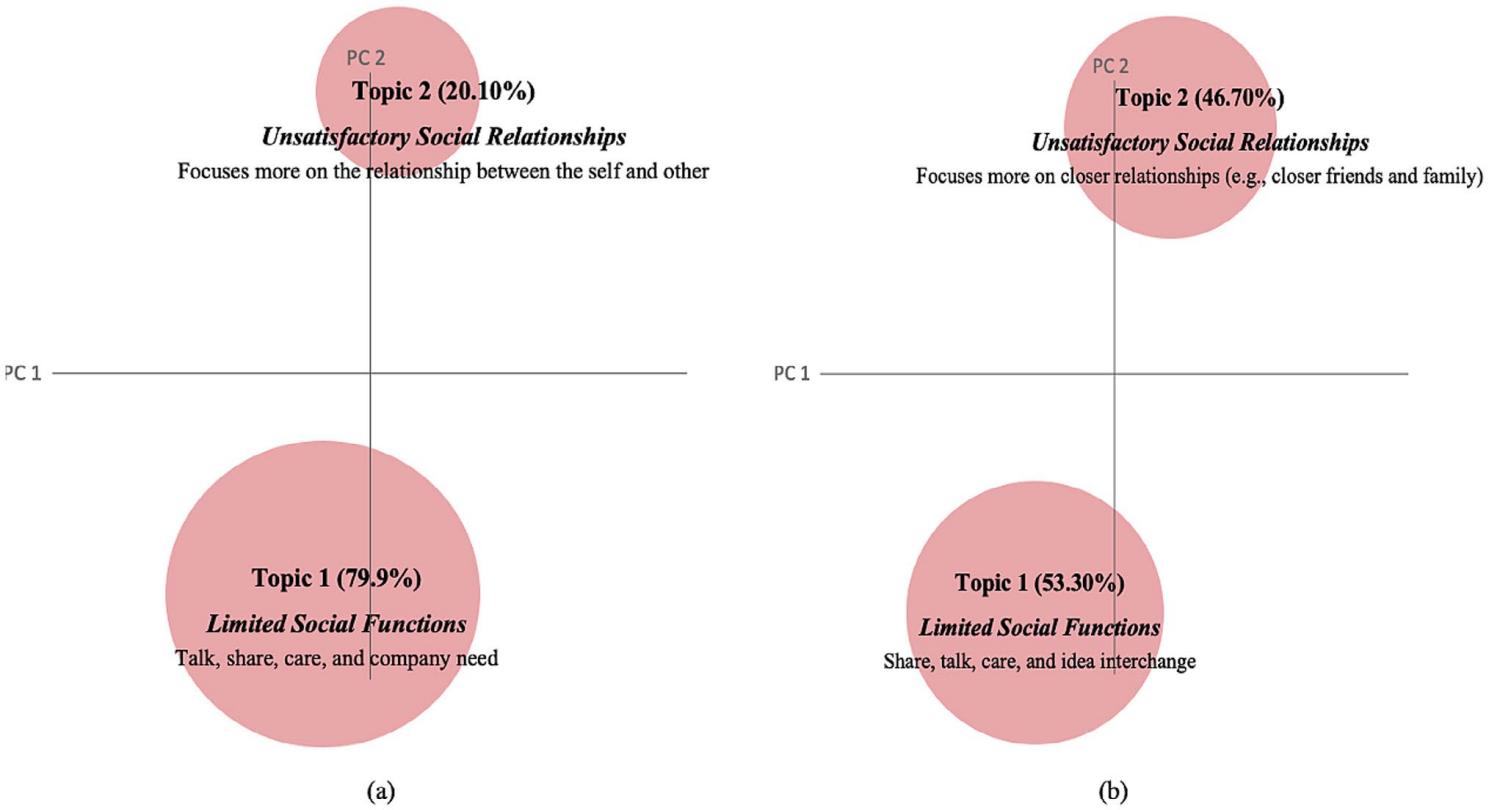
Inter‐topic distance map in the LDA model: (a) is the inter‐topic distance map of the younger age group, (b) is the inter‐topic distance map of the older age group. The default for computing inter‐topic distances is Jensen‐Shannon divergence, and for scaling the set of inter‐topic distances defaults to principal components (see Sievert and Shirley 2014). The areas of the circles are proportional to the relative prevalence of the topic in the corpus.

LDA model fit indices for different number of topics (*k*, ranging from one to six) for both the younger age group and the older age group is shown in Supporting Information S5. The Topic Coherence value reached its maximum at *k* = 2, and there was no semantic overlap when *k* = 2. Therefore, a two‐topic solution was selected as the optimal model for both the younger age group and the older age group. The visualization of the inter‐topic distances for the two groups was shown in Figure 5, where the area of each circle is proportional to the topic's prevalence in the corpus. The most representative words for each topic and the distribution probabilities of the words for each topic are listed in Supporting Information S5.

Although both groups manifested two shared themes, subtle yet crucial differences emerged in their specific semantic content. Topic 1 is *Limited Social Functions* (e.g., “Not being able to talk to people around me,” “Having nobody to turn to for a chat, meet up with, to confide in, share experiences with”). The younger age group predominantly focused on this topic, accounting for 79.90% of the younger age group's corpus, such as the lack of talk, sharing, and companionship. In contrast, the older age group showed a relatively lower focus on general social deficits (accounting for 53.30% of the older age group's corpus). While the older age group also perceived the lack of talking and sharing, they uniquely emphasized a lack of opportunities of a deep exchange of ideas. Topic 2 is *Unsatisfactory Social Relationships* (e.g., “I have friends but no best friend,” “Loneliness is the feeling of your heart jumping out of your chest when you worry about being alone without a partner or family”). In this topic, the older age group exhibited relatively less self‐centeredness compared to the younger age group. Instead, the older age group's narratives centered on their closest, most meaningful ties (e.g., spouses, children, long‐term friends). Moreover, the affection associated with loneliness was different between the two groups. The younger age group predominantly expressed anxiety (a high‐arousal negative emotion), and the older age group felt a sense of sadness (a low‐arousal negative emotion).

## 4 Discussion

Drawing upon the theory of gerotranscendence and the frameworks of stigma and meaning‐making (Bos et al. 2013; Martela and Steger 2016; Tornstam 2011), the current study employed polynomial regression and MG‐SEM to analyze the quantitative scale data, complemented by LDA to explore the textual data. This dual analytical approach was adopted to gain a more comprehensive and nuanced understanding of the age‐loneliness relationship and underlying factors.

### 4.1 Age and Loneliness

The current study advances the understanding of loneliness across the lifespan by empirically confirming an inverted *U*‐shaped relationship between age and loneliness. Specifically, a precise inflection point at 39.2 years of age was identified, indicating that loneliness steadily increases in the younger age group, peaks in midlife, and subsequently demonstrates a gradual decline into later life.

This non‐linear relationship provides nuanced support for age‐related emotional resilience (Charles 2010; Scheibe and Carstensen 2010). As the theory of gerotranscendence suggests, transcendence is a natural process that comes with aging (Tornstam 1997). For instance, social networks tend to narrow with age (Hajek and König 2020; Kim et al. 2023), and the quality of emotionally close social partners is more important for older individuals (Xing et al. 2017). As a result, older individuals may redefine social relationships and decrease the need for superficial social contact (Tornstam 1997), thus reducing the experience of loneliness. However, before the inflection point (in the younger age group), individuals often face intense social pressures regarding social network expansion, exacerbating the experience of loneliness (Qualter et al. 2015).

In summary, this finding calls for reconsidering the conventional perspective of the association between age and loneliness. It highlights that loneliness is not unique to older individuals and suggests that the finding of lower loneliness in older agewas observed in the current sample.

### 4.2 The Mediating Role of the Loneliness Self‐Stigma

Consistent with the hypothesis, loneliness self‐stigma was found to mediate the relationship between age and loneliness. Specifically, the older age group demonstrated lower loneliness self‐stigma and further experienced lower loneliness frequency, intensity, and duration. Corroborating the results of the mediation, younger participants expressed feelings of shame and embarrassment of loneliness in the free‐text question. For instance, they reported “Feeling ashamed of being lonely and uncomfortable admitting to my friends that I am lonely” (20 years old) and described “Being embarrassed of going alone” (36 years old).

In light of the theory of gerotranscendence (Tornstam 1997, 2011), the older age group demonstrated a significant decrease in self‐centeredness alongside an increase in self‐integration. This psychological maturation renders them less vulnerable to the perceived judgments of others, effectively buffering them against the self‐stigma associated with loneliness. Another possible explanation is that public awareness of loneliness is greatest for older individuals (Pitman et al. 2018), with much of the scientific research and social campaigns on loneliness being focused on older individuals (Chawla et al. 2021; Gerst‐Emerson and Jayawardhana 2015). Thus, loneliness is widely perceived as a more normative and socially acceptable experience in later life, thereby diminishing the stigmatization (Barreto et al. 2022). However, although the current study did not find a significant pathway from age to loneliness self‐stigma in the younger age group, previous studies suggested that younger individuals attach great importance to building and maintaining social relationships; they are more vigilant about personal evaluations from society and are particularly sensitive to accepting and associating themselves with stereotypes pertinent to loneliness (Tsai and Reis 2009; Zheng et al. 2023). Moreover, higher self‐stigma further increases loneliness (Qin et al. 2024). Because they fear embarrassment and of negative social evaluation, individuals experiencing higher loneliness self‐stigma suppress their emotions and avoid disclosing their feelings (Mental Health Foundation 2022). This reluctance to share can lead to greater emotional isolation and exacerbate loneliness (Ko et al. 2022; Prizeman et al. 2023).

### 4.3 The Mediating Role of the Meaning of Loneliness

Consistent with the hypothesis, the meaning of loneliness played a mediating role between age and loneliness. Specifically, the older age group assigned more positive than negative meanings to loneliness and experienced lower loneliness. The free‐text responses also revealed that some older participants framed loneliness with a more positive perspective. For example, they noted that it is “nice to spend ‘alone’ time” (74 years old) and stated “I am used to my own company and have a hobby” (70 years old).

According to the theory of gerotranscendence (Tornstam 1997, 2011), older individuals tend to reduce superficial connections, reconstructing cognitive strategies to reframe negative interpretations of loneliness (Carstensen and Reynolds 2023). This includes reaffirming meaning in areas where one feels vulnerable, such as reflecting on the negative meaning of loneliness (e.g., loss, being left out, and sadness), and redirecting focus toward discovering other potential aspects. For instance, older individuals consider loneliness as commonplace and focus on its benefits (Kharicha et al. 2021), such as freedom and self‐growth (Kvaal et al. 2014). This may enable older individuals to “open the door” of loneliness (Kitzmüller et al. 2018) and, thus, experience less loneliness. On the contrary, previous evidence indicates that younger individuals attach great importance to social networks (Moore and Leung 2002). When there is a gap between actual and ideal social relationships, younger individuals may ascribe the circumstance to undesirable meanings that ultimately link to loneliness.

### 4.4 The Three Dimensions of Loneliness in the Mediation Model

The finding suggests that loneliness is a multidimensional construct, including frequency, intensity, and duration. In addition to aligning with the two overarching topics (discussed in the next section), participants' free‐text answers also revealed nuanced dimensions of the loneliness experience. These included: loneliness frequency, with descriptions such as “Sometimes the expectation of company can be disappointing” (37 years old) and “I feel cut off from society sometimes” (42 years old); loneliness intensity, with statements ranging from “I still get very lonely” (25 years old) to “I am quite lonely, but not very lonely” (57 years old); loneliness duration, with participants mentioning specific timeframes like “Not speaking to anyone all day” (27 years old) or “Spending a lot of time alone” (27 years old).

Comparing the mediating effects among different dimensions of loneliness provided critical and unique information that may be obscured or confounded in previous literature. We found that the mediating effect of loneliness self‐stigma in the relationship between age and loneliness frequency/intensity was stronger than in the relationship between age and loneliness duration; additionally, the mediating effect of meaning of loneliness in the relationship between age and loneliness intensity was stronger than in the relationship between age and loneliness duration. These findings further imply that the gerotranscendent processes (e.g., diminished self‐stigma and enhanced positive meaning‐making in the context of the current study) protect older individuals against loneliness frequency and intensity, rather than attenuating its prolonged or chronic duration. One potential explanation is related to negative emotional sensitivity (Sauer‐Zavala et al. 2012). Cognitive factors (e.g., self‐stigma and negative meaning‐making) may heighten an individual's sensitivity to feelings of loneliness, with even brief moments of isolation potentially magnified into more frequent and intensive experiences of loneliness. These findings highlight the necessity of delineating loneliness into distinct dimensions, thereby guiding future research and enabling the design of tailored interventions for older individuals. For instance, strategies designed with a gerotranscendent perspective may prove efficacious in ameliorating frequent and severe loneliness.

### 4.5 The Textual Semantic Topics Towards Loneliness

The textual semantic topics towards loneliness are consistent across the younger and older age groups, while there are still discrepancies between the two age groups. First, although the two groups both experience the *Limited Social Functions* (e.g., the lack of talk and sharing with other people), the younger age group expressed more experiences in this topic compared with the older age group, and the older age group uniquely emphasized a lack of opportunities to exchange inner thoughts with others. Second, in terms of *Unsatisfactory Social Relationships*, older individuals tended to focus more on closer relationships. This finding was consistent with gerotranscendence (Tornstam 1997) and socioemotional selectivity theory (Carstensen et al. 1999): aging is accompanied by a decreased centrality on the self and a deliberate withdrawal from superficial relationships, favoring instead highly meaningful connections where deep ideas can be explored and understood. Third, the negative affective vocabulary accompanying loneliness shifted across the younger age group and the older age group. Specifically, high‐arousal negative emotion (e.g., anxiety) was predominantly expressed by the younger age group, while low‐arousal negative emotion (e.g., sadness) was expressed more by the older age group. This finding aligns with people's developmental stage. Social deficits threaten younger individuals' urgent need to build social capital, thereby triggering an anxious response. Conversely, the older individuals are not anxiously striving to build new social relationships; rather, the sadness reflects a mature, reflective acceptance of the irreversible loss of irreplaceable and deep relational figures (Charles 2010; Scheibe and Carstensen 2010).

Furthermore, the textual semantic findings in the LDA provided the phenomenological context to enrich the interpretation of the psychological mediators in MG‐SEM. On the one hand, younger individuals' heightened self‐focus and hypervigilance regarding social relationships increase vulnerability to social threat and loneliness stigma (Hawkley and Cacioppo 2010; Osborn et al. 2021). In contrast, the older individuals demonstrated a shift away from self‐focus and superficial validation (Tornstam 2005). By reducing the need to maintain a broad social network, older individuals escape from the social pressures and normative expectations. On the other hand, the younger age group expressed loneliness more as a deficit in social functioning, whereas the older age group particularly described it as the absence of profound intellectual and relational depth. By framing loneliness in a way that honors the value of irreplaceable, close social ties (Carstensen et al. 1999; Charles 2010) rather than an anxious experience, the older individuals transform it into a more meaningful existential state (Tornstam 2005).

### 4.6 Limitations and Future Directions

Several limitations should be addressed in future research. First, self‐reported data were inherently susceptible to the influence of social desirability bias. Although a preliminary Harman's single‐factor test indicated that common method bias was not a serious problem in the current study, this diagnostic tool cannot definitively rule out or isolate shared method variance (Podsakoff et al. 2003). Consequently, the magnitude of the observed structural associations should be interpreted with the understanding that relying exclusively on self‐reports may still partially inflate some relationships. Integrating neurophysiological indicators (Franco‐O'Byrne et al. 2023) and more advanced diagnostic tools for detecting methodological biases (Podsakoff et al. 2024) could further enhance the interpretability and generalizability of the findings.

Second, the reliance on an online survey introduces self‐selection biases. On the one hand, people with better physical and mental health, higher educational attainment, greater digital literacy, and a pre‐existing interest in loneliness‐related topics were more likely to participate in the study. Accordingly, these selection processes may systematically exclude the most vulnerable or technologically disadvantaged older individuals. The demographic composition was also predominantly female and concentrated in the United Kingdom. Although additional analyses (after considering the gender and cultural background) yielded results consistent with the primary findings, these imbalances limited the generalizability of the conclusions. On the other hand, while the age‐stratified MG‐SEM based on a non‐linear relationship yielded theoretical insights into developmental transformations, the group sample sizes were unequal in the current study. Although the absolute sample size within each derived subgroup remained relatively large for adequate statistical power in SEM (Kline 2023), the demographic imbalance is noted. Future research utilizing nationally representative and balanced sampling for age, gender, and cultural background is recommended to rigorously cross‐validate and extend the mechanisms identified in this study.

Third, conceptual complexities are inherent in measuring multidimensional psychological constructs (e.g., different subdimensions of meaning and loneliness). For instance, as with many checklist‐style inventories, summing scores of the meaning of loneliness (i.e., summing positive and reverse‐coded negative items) may simplify the construct. Future research should aim to unpack this complexity by utilizing person‐centered methodologies, such as Latent Profile Analysis (LPA) and Latent Transition Analysis (LTA), to identify distinct profiles and how these profiles shift across the lifespan.

Fourth, the cross‐sectional nature of the data precludes further definitive conclusions regarding causality or intra‐individual developmental change. It is difficult to isolate pure developmental maturation (i.e., getting older) from the unique generational environments in which different cohorts were socialized. Longitudinal panel designs and experience sampling methodology (Costa and McCrae 1982; Myin‐Germeys et al. 2018) should be employed to track individuals' stigma and meaning‐making towards loneliness as they age.

Lastly, while LDA provides an unsupervised machine learning approach for extracting semantic topics from large‐scale text, future research should complement LDA with in‐depth qualitative interviews (Sholokhova et al. 2022). The phenomenological approaches are needed to compare and understand the nuanced experiences of loneliness‐related stigmatization and meaning‐making between younger and older age groups, informing the development of more targeted, age‐specific psychological interventions.

## 5 Conclusion and Implications

This study further enriched the theory of gerotranscendence in the loneliness domain with the perspective of stigma and meaning‐making. First, the findings empirically confirmed a non‐linear, inverted *U*‐shaped relationship between age and loneliness, identifying a precise inflection at 39.2 years of age. Second, we found that the older age group experienced lower overall loneliness due to two parallel gerotranscendent mechanisms: a reduction in loneliness self‐stigma and an enhanced capacity for positive meaning‐making. Third, the above mediations demonstrated dimensional specificity for loneliness, which primarily attenuated the frequency and acute intensity of loneliness rather than its chronic duration. Fourth, corroborated by the textual semantic topics extracted via LDA, the study further captured the nuanced, age‐specific variations in the lived experience of loneliness.

These findings suggest potential practical interventions for addressing loneliness. While increasing social interaction and providing social skills training have been shown to alleviate loneliness (Pearce et al. 2021), our results indicate it may be beneficial to target internal psychological factors. Mental health professionals should incorporate stigma‐reduction and meaning‐making approaches into therapeutic frameworks to help individuals re‐frame loneliness in a more constructive light (Rodriguez et al. 2025). Public campaigns and media narratives should aim to destigmatize loneliness by reframing it as a shared human experience with opportunities for personal growth and connection. In addition, we should note people's preconceived notions about a group (e.g., older individuals are bound to be lonely, while younger individuals are bound to be sociable) and alleviate stigmatization. Collectively, these findings emphasize the value of understanding loneliness through a gerotranscendent lens that considers psychological mechanisms, individual experiences, and social attitudes. Together the findings of self‐stigma, meaning‐making and age related differences in loneliness may inform the development of more effective interventions tailored to different age groups.

## Funding

Wellcome Trust [Funder reference: 209625/Z/17/Z] funded the BBC Loneliness Experiment, awarded to Pamela Qualter, Manuela Barreto, and Christina Victor. This paper was also supported by the National Natural Science Foundation of China [Funder reference: 32271136] to Guangyu Zhou.

## Ethics Statement

This study received ethical approval from the University of Manchester (Reference No.: 2017‐2710‐4594), and participants gave their informed consent before participating in this study.

## Conflicts of Interest

The authors declare no conflicts of interest.

## Supporting information


**Table S1:** Characteristics of participants.
**Table S2:** Between‐group differences of the mediating effects.
**Table S3:** The model fit of the 2‐group MG‐SEM controlling for IDV.
**Table S4:** Parallel mediating effects of loneliness self‐stigma and meaning of loneliness in Group 1 (younger adults, <= 39.2).
**Table S5:** Parallel mediating effects of loneliness self‐stigma and meaning of loneliness in Group 2 (older adults, > 39.2).
**Table S6:** The model fit of the 4‐group MG‐SEM.
**Table S7:** Parallel mediating effects of loneliness self‐stigma and meaning of loneliness in Group 1 (younger female, <= 39.2).
**Table S8:** Parallel mediating effects of loneliness self‐stigma and meaning of loneliness in Group 2 (younger male, <= 39.2).
**Table S9:** Parallel mediating effects of loneliness self‐stigma and meaning of loneliness in Group 3 (older female, > 39.2).
**Table S10:** Parallel mediating effects of loneliness self‐stigma and meaning of loneliness in Group 4 (older male, > 39.2).
**Table S11:** The model fit of the 4‐group MG‐SEM.
**Table S12:** Parallel mediating effects of loneliness self‐stigma and meaning of loneliness in Group 1 (emerging adulthood, <= 25 years).
**Table S13:** Parallel mediating effects of loneliness self‐stigma and meaning of loneliness in Group 2 (early‐middle adulthood, 25~44 years).
**Table S14:** Parallel mediating effects of loneliness self‐stigma and meaning of loneliness in Group 3 (later‐middle adulthood, 45~64 years).
**Table S15:** Parallel mediating effects of loneliness self‐stigma and meaning of loneliness in Group 4 (later adulthood, > = 65 years).
**Table S16:** The model fit and mediating effects of competing sequential (chain) mediation models.
**Table S17:** The LDA model index of topic number in the younger age group (<= 39.2 years old).
**Table S18:** The LDA model index of topic number in the older age group (> 39.2 years old).
**Figure S1:** Top‐20 most relevant words (terms) for each topic of the younger age group (<= 39.2 years old).
**Figure S2:** Top‐20 most relevant words (terms) for each topic of the older age group (> 39.2 years old).
**Table S19:** The words and their distribution probabilities for each topic.

## Data Availability

Access to the dataset is available from the authors upon reasonable request.
