# Supplementary material for: Roles of Self‐Stigma and Meaning in the Relationship Between Age and Loneliness: A Perspective From Gerotranscendence
Source: Psych J. 2026 Jul 26;15(4):e70115. doi: 10.1002/pchj.70115 (PMC13402103; doi:10.1002/pchj.70115)
Supplement: Supplementary file 1 — Table S1: Characteristics of participants. Table S2: Between‐group differences of the mediating effects. Table S3: The model fit of the 2‐group MG‐SEM controlling for IDV. Table S4: Parallel mediating effects of loneliness self‐stigma and meaning of loneliness in Group 1 (younger adults, <= 39.2). Table S5: Parallel mediating effects of loneliness self‐stigma and meaning of loneliness in Group 2 (older adults, > 39.2). Table S6: The model fit of the 4‐group MG‐SEM. Table S7: Parallel mediating effects of loneliness self‐stigma and meaning of loneliness in Group 1 (younger female, <= 39.2). Table S8: Parallel mediating effects of loneliness self‐stigma and meaning of loneliness in Group 2 (younger male, <= 39.2). Table S9: Parallel mediating effects of loneliness self‐stigma and meaning of loneliness in Group 3 (older female, > 39.2). Table S10: Parallel mediating effects of loneliness self‐stigma and meaning of loneliness in Group 4 (older male, > 39.2). Table S11: The model fit of the 4‐group MG‐SEM. Table S12: Parallel mediating effects of loneliness self‐stigma and meaning of loneliness in Group 1 (emerging adulthood, <= 25 years). Table S13: Parallel mediating effects of loneliness self‐stigma and meaning of loneliness in Group 2 (early‐middle adulthood, 25~44 years). Table S14: Parallel mediating effects of loneliness self‐stigma and meaning of loneliness in Group 3 (later‐middle adulthood, 45~64 years). Table S15: Parallel mediating effects of loneliness self‐stigma and meaning of loneliness in Group 4 (later adulthood, > = 65 years). Table S16: The model fit and mediating effects of competing sequential (chain) mediation models. Table S17: The LDA model index of topic number in the younger age group (<= 39.2 years old). Table S18: The LDA model index of topic number in the older age group (> 39.2 years old). Figure S1: Top‐20 most relevant words (terms) for each topic of the younger age group (<= 39.2 years old). Figure S2: Top‐20 most relevant words (terms) [file PCHJ-15-e70115-s001.docx]

**Supplementary material**

Contents

[Supplementary 1: Characteristics of participants 2](#_Toc231656500)

[Supplementary 2: Dimensionality and measurement validation of loneliness 4](#_Toc231656501)

[Supplementary 3: Between-group differences of the mediating effects 6](#_Toc231656502)

[Supplementary 4: Robustness and sensitivity analyses 7](#_Toc231656503)

[Supplementary 5: LDA 22](#_Toc231656504)

# **Supplementary 1: Characteristics of participants**

Table S1. *Characteristics of participants.*

|  | *n* | *M*(*SD*) |
| --- | --- | --- |
| **Demographical factors** |  |  |
| Age |  |  |
| 16-25 | 595 | 21.47(0.11) |
| 26-35 | 922 | 30.50(0.09) |
| 36-45 | 1093 | 40.72(0.09) |
| 46-55 | 1585 | 50.82(0.07) |
| 56-65 | 1489 | 60.27(0.07) |
| higher than 65 | 1191 | 70.96(0.14) |
| Gender |  |  |
| Female | 4661 |  |
| Male | 2214 |  |
| **SES factors** |  |  |
| SSS |  | 6.03(1.82) |
| Income met (7 missing) |  |  |
| very well | 1180 |  |
| fairly well | 3454 |  |
| Poorly | 2234 |  |
| Educational years |  | 17.42(5.01) |
| Employment (9 missing) |  |  |
| not active in labor marked | 2345 |  |
| active in labor marked | 4521 |  |
| **Other loneliness factors** |  |  |
| Solitude |  | 5.12(1.81) |
| Living alone (3 missing) |  |  |
| No | 3976 |  |
| Yes | 2896 |  |

*Note*. SES = Socioeconomic Status; SSS = Subjective Social Status.

# **Supplementary 2: Dimensionality and measurement validation of loneliness**

To empirically justify the decomposition of loneliness into three distinct dimensions (frequency, intensity, and duration) and to rule out potential measurement redundancy, we conducted a series of Confirmatory Factor Analyses (CFAs) and a bifactor analysis prior to estimating the primary structural models.

1. One-Factor vs. Three-Factor Comparison

First, compared a unidimensional (1-factor) model, where all items loaded onto a single global loneliness construct, against the hypothesized 3-factor model. The 3-factor model demonstrated superior fit to the data (χ^2^/*df* = 3.59, CFI = 0.88, TLI = 0.85, SRMR = 0.03, and RMSEA = 0.02) compared to the 1-factor model (χ^2^/*df* = 4.51, CFI = 0.80, TLI = 0.83, SRMR = .04, and RMSEA = 0.02). Second, a Satorra-Bentler scaled Likelihood Ratio Test (LRT) was conducted (ΔS-Bχ^2^; Satorra & Bentler, 2001). The results confirmed that the 3-factor model fits the data significantly better than the 1-factor solution (*∆*χ^2^ = 21.26, *p* < 0.001), and the improvement in the Comparative Fit Index (*∆*CFI = 0.08) substantially exceeded the established methodological threshold of 0.02 for demonstrating meaningful structural improvement (Chen, 2007; Cheung & Rensvold, 2002).

1. Bifactor Modeling

To further partition the variance and assess whether the items predominantly reflect a single underlying trait or distinct sub-dimensions, a bifactor model was estimated. Methodological guidelines suggest that a composite score can be considered essentially unidimensional if the general factor’s (g) Omega Hierarchical (ω) value exceeds .70 or .80 (Reise et al., 2013; Rodriguez et al., 2016a, 2016b).

In the current sample, the general loneliness factor (g) yielded an ω of 0.46, falling substantially below the threshold for unidimensionality. This indicates that the item-level variance is not driven solely by a generalized loneliness factor. While the loneliness specific factor’s (s) average ω (sub-dimensions) was 0.50, which was higher than the g-factor ω, suggesting the differentiation of sub-dimensions. After controlling for the general factor, the s-factor ω for *duration* (ω = 0.61) and *intensity* (ω = 0.67) demonstrated exceptionally high unique variance, well above the acceptable minimums. While the *frequency* dimension exhibited relatively lower unique variance (ω = 0.20; it should be typically > 0.20, Smits et al., 2014), which is theoretically expected, as most global loneliness items inherently capture frequency (e.g., original UCLA Loneliness Scale, Russell et al., 1980; Emotional and Social Loneliness Scale, Vincenzi & Grabosky, 1987), the overall bifactor indices support the necessity of separating several factors to capture distinct, non-redundant psychometric information.

# **Supplementary 3: Between-group differences of the mediating effects**

To evaluate between-group differences in the mediating effects, the custom contrasts were specified to calculate the exact effect size differences between groups with a 5000-resample bootstrapping procedure.

The contrast analyses indicated that the mediating effects were significantly stronger in the older age group compared to the younger group. The results for all six contrast tests (i.e., the group differences for the mediating effects of loneliness self-stigma between age and loneliness frequency, intensity, and duration, separately; the group differences for the mediating effects of meaning of loneliness between age and loneliness frequency, intensity, and duration, separately).

Table S2. *Between-group differences of the mediating effects*

| Contrast |  |  | *β* | *SE* | *p* | *Boot LLCI* | *Boot ULCI* |
| --- | --- | --- | --- | --- | --- | --- | --- |
| Age → | Loneliness self-stigma | → Loneliness frequency | -0.042 | 0.007 | < 0.001 | -0.057 | -0.028 |
|  |  | → Loneliness intensity | -0.050 | 0.008 | < 0.001 | -0.066 | -0.034 |
|  |  | → Loneliness duration | -0.041 | 0.007 | < 0.001 | -0.055 | -0.027 |
| Age → | Meaning of loneliness | → Loneliness frequency | -0.139 | 0.021 | < 0.001 | -0.180 | -0.099 |
|  |  | → Loneliness intensity | -0.141 | 0.021 | < 0.001 | -0.182 | -0.101 |
|  |  | → Loneliness duration | -0.125 | 0.018 | < 0.001 | -0.161 | -0.089 |

*Note.* The contrast is the mediating effect in the older age group minus the corresponding indirect effect in the younger age group. Adjusting for gender, SES, living alone, and solitude.

# **Supplementary 4: Robustness and sensitivity analyses**

Given the self-selected nature of the online dataset, which features a strong geographic concentration in the United Kingdom and a notable gender imbalance (a higher proportion of females), four additional analyses were conducted.

**(1) For UK representation skew:** Because the current sample is predominantly UK-based, it is important to ensure that the findings are not merely artifacts of Western, highly individualistic culture regarding loneliness. To control for macro-level cultural variance, we integrated Hofstede’s Individualism Index (IDV; Hofstede, 1997). This index 101 countries on a 100-point scale ranging from highly collectivistic (e.g., Guatemala = 6) to highly individualistic (e.g., United States = 91, United Kingdom = 89). Each participant was assigned an IDV score (Hofstede’s Individualism Index, ranging from 6 to 91) based on their country of residence. The Z-score of this continuous cultural index was included as one of the covariates in the MG-SEM. The structural coefficients and indirect effects remained virtually identical to the models without controlling for the IDV.

The inclusion of the IDV did not alter the primary findings, suggesting the results of the current study remain robust across different individualistic cultural contexts.

Table S3. *The model fit of the 2-group MG-SEM controlling for IDV*

|  | *n* | *χ^2^/df* | *p* | *AIC* | *BIC* | *CFI* | *TLI* | *SRMR* | *RMSEA* |
| --- | --- | --- | --- | --- | --- | --- | --- | --- | --- |
| Group 1 (Younger adults, <= 39.2) | 1922 | 8.68 | < 0.001 | 28780.56 | 29086.43 | 0.99 | 0.95 | 0.06 | 0.02 |
| Group 2 (Older adults, > 39.2) | 4953 | 10.32 | < 0.001 | 74688.41 | 75046.34 | 0.99 | 0.99 | 0.05 | 0.02 |

Table S4. *Parallel mediating effects of Loneliness self-stigma and Meaning of loneliness in Group 1 (Younger adults, <= 39.2)*

| Pathway |  |  | *β* | *SE* | *p* | *Boot LLCI* | *Boot ULCI* |
| --- | --- | --- | --- | --- | --- | --- | --- |
| **Indirect effects** | |  |  |  |  |  |  |
| Age → | Loneliness self-stigma | → Loneliness frequency | 0.005 | 0.028 | 0.500 | -0.036 | 0.073 |
|  |  | → Loneliness intensity | 0.005 | 0.008 | 0.500 | -0.039 | 0.079 |
|  |  | → Loneliness duration | 0.004 | 0.027 | 0.500 | -0.034 | 0.070 |
| Total indirect effect of Loneliness self-stigma | | | 0.014 | 0.022 | 0.500 | -0.109 | 0.223 |
| Age → | Meaning of loneliness | → Loneliness frequency | -0.007 | 0.005 | 0.426 | -0.014 | 0.006 |
|  |  | → Loneliness intensity | -0.008 | 0.006 | 0.420 | -0.018 | 0.007 |
|  |  | → Loneliness duration | -0.006 | 0.004 | 0.432 | -0.109 | 0.005 |
| Total indirect effect of Meaning of loneliness | | | -0.021 | 0.015 | 0.422 | -0.043 | 0.018 |
| **Total effects** | |  |  |  |  |  |  |
|  |  |  | -0.079 | 0.181 | 0.781 | -0.305 | 0.406 |

*Note.* Adjusting for gender, SES, living alone, solitude, and Hofstede’s Individualism Index.

Table S5. *Parallel mediating effects of Loneliness self-stigma and Meaning of loneliness in Group 2 (Older adults, > 39.2)*

| Pathway |  |  | *β* | *SE* | *p* | *Boot LLCI* | *Boot ULCI* |
| --- | --- | --- | --- | --- | --- | --- | --- |
| **Indirect effects** | |  |  |  |  |  |  |
| Age → | Loneliness self-stigma | → Loneliness frequency | -0.133 | 0.017 | < 0.001 | -0.166 | -0.099 |
|  |  | → Loneliness intensity | -0.134 | 0.017 | < 0.001 | -0.168 | -0.100 |
|  |  | → Loneliness duration | -0.119 | 0.016 | < 0.001 | -0.150 | -0.089 |
| Total indirect effect of Loneliness self-stigma | | | -0.386 | 0.049 | < 0.001 | -0.483 | -0.289 |
| Age → | Meaning of loneliness | → Loneliness frequency | -0.067 | 0.006 | < 0.001 | -0.079 | -0.055 |
|  |  | → Loneliness intensity | -0.058 | 0.006 | < 0.001 | -0.069 | -0.047 |
|  |  | → Loneliness duration | -0.056 | 0.006 | < 0.001 | -0.068 | -0.045 |
| Total indirect effect of Meaning of loneliness | | | -0.182 | 0.017 | < 0.001 | -0.215 | -0.148 |
| **Total effects** | |  |  |  |  |  |  |
|  |  |  | -0.717 | 0.058 | < 0.001 | -0.831 | -0.602 |

*Note.* Adjusting for gender, SES, living alone, solitude, and Hofstede’s Individualism Index.

**(2) For gender imbalance:** To ensure the higher proportion of female participants did not artificially drive the observed results, we expanded the current Multi-Group SEM. We stratified the sample into four groups based on both the inflection age point and gender (i.e., Younger Males, Younger Females, Older Males, Older Females).

Before the multi-group structural equation modeling (MG-SEM), measurement invariance across the four demographic groups was tested. The constrained metric invariance model showed no significant decrement in model fit compared to the configural invariance model (*∆*χ2 = 4.75, *p* = 0.576), indicating that the latent constructs carried identical meanings across age and gender groups.

The results indicated no substantive differences in the direction, statistical significance, or relative magnitude of the indirect effects between the gender-stratified 4-group model and the original age-only 2-group model, suggesting that the gerotranscendent mechanisms operate similarly for both men and women, despite the female-skewed sample.

Table S6. *The model fit of the 4-group MG-SEM*

|  | *n* | *χ2/df* | *p* | *AIC* | *BIC* | *CFI* | *TLI* | *SRMR* | *RMSEA* |
| --- | --- | --- | --- | --- | --- | --- | --- | --- | --- |
| Group 1 (Younger female, <= 39.2) | 1282 | 6.09 | < 0.001 | 18463.952 | 18721.76 | 0.99 | 0.95 | 0.06 | 0.02 |
| Group 2 (Younger male, <= 39.2) | 640 | 2.58 | < 0.010 | 9616.337 | 9839.41 | 0.99 | 0.96 | 0.05 | 0.02 |
| Group 3 (Older female, > 39.2) | 3379 | 6.38 | < 0.001 | 50046.318 | 50352.58 | 0.99 | 0.99 | 0.04 | 0.01 |
| Group 4 (Older male, > 39.2) | 1574 | 5.16 | < 0.001 | 23563.432 | 23831.5 | 0.99 | 0.99 | 0.05 | 0.02 |

Table S7. *Parallel mediating effects of Loneliness self-stigma and Meaning of loneliness in Group 1 (Younger female, <= 39.2)*

| Pathway |  |  | *β* | *SE* | *p* | *Boot LLCI* | *Boot ULCI* |
| --- | --- | --- | --- | --- | --- | --- | --- |
| Indirect effects | |  |  |  |  |  |  |
| Age → | Loneliness self-stigma | → Loneliness frequency | 0.022 | 0.022 | 0.325 | -0.021 | 0.065 |
|  |  | → Loneliness intensity | -0.060 | 0.008 | 0.324 | -0.02 | 0.069 |
|  |  | → Loneliness duration | -0.050 | 0.007 | 0.324 | -0.011 | 0.061 |
| Total indirect effect of Loneliness self-stigma | | | -0.088 | 0.022 | 0.324 | -0.064 | 0.195 |
| Age → | Meaning of loneliness | → Loneliness frequency | -0.009 | 0.003 | 0.335 | -0.014 | 0.005 |
|  |  | → Loneliness intensity | -0.006 | 0.004 | 0.320 | -0.018 | 0.006 |
|  |  | → Loneliness duration | -0.003 | 0.004 | 0.362 | -0.011 | 0.004 |
| Total indirect effect of Meaning of loneliness | | | -0.190 | 0.010 | 0.329 | -0.043 | 0.014 |
| Total effects | |  |  |  |  |  |  |
|  |  |  | -0.440 | 0.050 | 0.947 | -0.362 | 0.338 |

*Note.* Adjusting for SES, living alone, and solitude.

Table S8. *Parallel mediating effects of Loneliness self-stigma and Meaning of loneliness in Group 2 (Younger male, <= 39.2)*

| Pathway |  |  | *β* | *SE* | *p* | *Boot LLCI* | *Boot ULCI* |
| --- | --- | --- | --- | --- | --- | --- | --- |
| Indirect effects | |  |  |  |  |  |  |
| Age → | Loneliness self-stigma | → Loneliness frequency | -0.036 | 0.035 | 0.309 | -0.104 | 0.033 |
|  |  | → Loneliness intensity | -0.029 | 0.029 | 0.310 | -0.085 | 0.027 |
|  |  | → Loneliness duration | -0.025 | 0.025 | 0.310 | -0.074 | 0.024 |
| Total indirect effect of Loneliness self-stigma | | | -0.090 | 0.088 | 0.308 | -0.263 | 0.083 |
| Age → | Meaning of loneliness | → Loneliness frequency | -0.009 | 0.011 | 0.428 | -0.031 | 0.013 |
|  |  | → Loneliness intensity | -0.011 | 0.013 | 0.421 | -0.036 | 0.015 |
|  |  | → Loneliness duration | -0.010 | 0.013 | 0.425 | -0.035 | 0.015 |
| Total indirect effect of Meaning of loneliness | | | -0.030 | 0.037 | 0.422 | -0.102 | 0.043 |
| Total effects | |  |  |  |  |  |  |
|  |  |  | -0.313 | 0.247 | 0.205 | -0.797 | 0.171 |

*Note.* Adjusting for SES, living alone, and solitude.

Table S9. *Parallel mediating effects of Loneliness self-stigma and Meaning of loneliness in Group 3 (Older female, > 39.2)*

| Pathway |  |  | *β* | *SE* | *p* | *Boot LLCI* | *Boot ULCI* |
| --- | --- | --- | --- | --- | --- | --- | --- |
| **Indirect effects** | |  |  |  |  |  |  |
| Age → | Loneliness self-stigma | → Loneliness frequency | -0.113 | 0.012 | < 0.001 | -0.137 | -0.089 |
|  |  | → Loneliness intensity | -0.116 | 0.013 | < 0.001 | -0.141 | -0.09 |
|  |  | → Loneliness duration | -0.101 | 0.011 | < 0.001 | -0.124 | -0.079 |
| Total indirect effect of Loneliness self-stigma | | | -0.330 | 0.036 | < 0.001 | -0.400 | -0.26 |
| Age → | Meaning of loneliness | → Loneliness frequency | -0.044 | 0.007 | < 0.001 | -0.056 | -0.031 |
|  |  | → Loneliness intensity | -0.056 | 0.007 | < 0.001 | -0.07 | -0.041 |
|  |  | → Loneliness duration | -0.045 | 0.007 | < 0.001 | -0.058 | -0.031 |
| Total indirect effect of Meaning of loneliness | | | -0.144 | 0.02 | < 0.001 | -0.183 | -0.106 |
| **Total effects** | |  |  |  |  |  |  |
|  |  |  | -0.717 | 0.072 | < 0.001 | -0.858 | -0.576 |

*Note.* Adjusting for SES, living alone, and solitude.

Table S10. *Parallel mediating effects of Loneliness self-stigma and Meaning of loneliness in Group 4 (Older male, > 39.2)*

| Pathway |  |  | *β* | *SE* | *p* | *Boot LLCI* | *Boot ULCI* |
| --- | --- | --- | --- | --- | --- | --- | --- |
| **Indirect effects** | |  |  |  |  |  |  |
| Age → | Loneliness self-stigma | → Loneliness frequency | -0.162 | 0.015 | < 0.001 | -0.192 | -0.132 |
|  |  | → Loneliness intensity | -0.159 | 0.015 | < 0.001 | -0.189 | -0.130 |
|  |  | → Loneliness duration | -0.145 | 0.016 | < 0.001 | -0.176 | -0.114 |
| Total indirect effect of Loneliness self-stigma | | | -0.467 | 0.043 | < 0.001 | -0.551 | -0.382 |
| Age → | Meaning of loneliness | → Loneliness frequency | -0.065 | 0.010 | < 0.001 | -0.084 | -0.046 |
|  |  | → Loneliness intensity | -0.060 | 0.010 | < 0.001 | -0.080 | -0.041 |
|  |  | → Loneliness duration | -0.053 | 0.010 | < 0.001 | -0.072 | -0.034 |
| Total indirect effect of Meaning of loneliness | | | -0.178 | 0.028 | < 0.001 | -0.233 | -0.124 |
| **Total effects** | |  |  |  |  |  |  |
|  |  |  | -0.707 | 0.100 | < 0.001 | -0.902 | -0.511 |

*Note.* Adjusting for SES, living alone, and solitude.

**(3) additional Multi-Group SEM across theoretically defined adulthood**

An additional Multi-Group Structural Equation Model (MG-SEM) was conducted based on theoretically defined adulthood: emerging adulthood (<= 25 years), early-middle adulthood (25~44), later-middle adulthood (45~64), and older adulthood (>= 65 years). (Arnett, 2000; Morbey et al., 2025).

Before estimating the structural paths, measurement invariance across the four distinct age groups was tested to ensure the psychological constructs were interpreted consistently. The constrained metric invariance model showed no significant decrement in model fit compared to the configural invariance model (*∆*χ2 = 2.034, *p* = 0.92), satisfying the prerequisite for multi-group structural comparisons.

Consistent with the primary findings based on the empirical inflection age point, the emerging adulthood group did not demonstrate indirect effects through self-stigma or the meaning of loneliness. In contrast, the older adulthood group demonstrated parallel mediating effects.

Table S11. *The model fit of the 4-group MG-SEM*

|  | *n* | *χ2/df* | *p* | *AIC* | *BIC* | *CFI* | *TLI* | *SRMR* | *RMSEA* |
| --- | --- | --- | --- | --- | --- | --- | --- | --- | --- |
| Group 1 (Emerging adulthood, <= 25) | 595 | 4.36 | < 0.001 | 7572.634 | 7831.559 | 0.96 | 0.89 | 0.076 | 0.043 |
| Group 2 (Early-middle adulthood, 25~44) | 1895 | 11.31 | < 0.001 | 23449.540 | 23776.812 | 0.96 | 0.91 | 0.074 | 0.041 |
| Group 3 (Later-middle adulthood, 45~64) | 3194 | 19.00 | < 0.001 | 41168.836 | 41526.909 | 0.97 | 0.92 | 0.075 | 0.042 |
| Group 4 (Later adulthood, >= 65) | 1191 | 9.60 | < 0.001 | 18072.248 | 18370.21 | 0.98 | 0.90 | 0.075 | 0.050 |

Table S12. *Parallel mediating effects of Loneliness self-stigma and Meaning of loneliness in Group 1 (Emerging adulthood,* <= 25 years*)*

| Pathway |  |  | *β* | *SE* | *p* | *Boot LLCI* | *Boot ULCI* |
| --- | --- | --- | --- | --- | --- | --- | --- |
| **Indirect effects** | |  |  |  |  |  |  |
| Age → | Loneliness self-stigma | → Loneliness frequency | 0.097 | 0.099 | 0.326 | -0.097 | 0.292 |
|  |  | → Loneliness intensity | 0.086 | 0.088 | 0.328 | -0.086 | 0.257 |
|  |  | → Loneliness duration | 0.079 | 0.081 | 0.329 | -0.079 | 0.237 |
| Total indirect effect of Loneliness self-stigma | | | 0.262 | 0.267 | 0.327 | -0.262 | 0.785 |
| Age → | Meaning of loneliness | → Loneliness frequency | 0.003 | 0.015 | 0.846 | -0.026 | 0.032 |
|  |  | → Loneliness intensity | 0.005 | 0.026 | 0.845 | -0.045 | 0.056 |
|  |  | → Loneliness duration | 0.002 | 0.012 | 0.846 | -0.021 | 0.025 |
| Total indirect effect of Meaning of loneliness | | | 0.010 | 0.052 | 0.845 | -0.092 | 0.113 |
| **Total effects** | |  |  |  |  |  |  |
|  |  |  | -1.446 | 0.551 | 0.010 | -2.527 | -0.366 |

*Note.* Adjusting for gender, SES, living alone, and solitude.

Table S13. *Parallel mediating effects of Loneliness self-stigma and Meaning of loneliness in Group 2 (Early-middle adulthood,* 25~44 years*)*

| Pathway |  |  | *β* | *SE* | *p* | *Boot LLCI* | *Boot ULCI* |
| --- | --- | --- | --- | --- | --- | --- | --- |
| **Indirect effects** | |  |  |  |  |  |  |
| Age → | Loneliness self-stigma | → Loneliness frequency | 0.021 | 0.022 | 0.337 | -0.022 | 0.064 |
|  |  | → Loneliness intensity | 0.022 | 0.023 | 0.337 | -0.023 | 0.067 |
|  |  | → Loneliness duration | 0.019 | 0.019 | 0.335 | -0.019 | 0.056 |
| Total indirect effect of Loneliness self-stigma | | | 0.062 | 0.064 | 0.336 | -0.064 | 0.187 |
| Age → | Meaning of loneliness | → Loneliness frequency | -0.016 | 0.007 | 0.026 | -0.029 | -0.002 |
|  |  | → Loneliness intensity | -0.017 | 0.008 | 0.023 | -0.032 | -0.002 |
|  |  | → Loneliness duration | -0.014 | 0.006 | 0.026 | -0.027 | -0.002 |
| Total indirect effect of Meaning of loneliness | | | -0.047 | 0.021 | 0.023 | -0.088 | -0.007 |
| **Total effects** | |  |  |  |  |  |  |
|  |  |  | 0.440 | 0.164 | 0.007 | 0.119 | 0.762 |

*Note.* Adjusting for gender, SES, living alone, and solitude.

Table S14. *Parallel mediating effects of Loneliness self-stigma and Meaning of loneliness in Group 3 (Later-middle adulthood,* 45~64 years*)*

| Pathway |  |  | *β* | *SE* | *p* | *Boot LLCI* | *Boot ULCI* |
| --- | --- | --- | --- | --- | --- | --- | --- |
| **Indirect effects** | |  |  |  |  |  |  |
| Age → | Loneliness self-stigma | → Loneliness frequency | -0.112 | 0.029 | < 0.001 | -0.170 | -0.055 |
|  |  | → Loneliness intensity | -0.112 | 0.030 | < 0.001 | -0.172 | -0.053 |
|  |  | → Loneliness duration | -0.098 | 0.026 | < 0.001 | -0.149 | -0.048 |
| Total indirect effect of Loneliness self-stigma | | | -0.323 | 0.085 | < 0.001 | -0.490 | -0.156 |
| Age → | Meaning of loneliness | → Loneliness frequency | -0.031 | 0.010 | 0.001 | -0.051 | -0.012 |
|  |  | → Loneliness intensity | -0.040 | 0.011 | < 0.001 | -0.061 | -0.018 |
|  |  | → Loneliness duration | -0.032 | 0.009 | 0.001 | -0.050 | -0.013 |
| Total indirect effect of Meaning of loneliness | | | -0.103 | 0.030 | 0.001 | -0.161 | -0.044 |
| **Total effects** | |  |  |  |  |  |  |
|  |  |  | -0.705 | 0.132 | < 0.001 | -0.963 | -0.447 |

*Note.* Adjusting for gender, SES, living alone, and solitude.

Table S15. *Parallel mediating effects of Loneliness self-stigma and Meaning of loneliness in Group 4 (Later adulthood,* >= 65 years*)*

| Pathway |  |  | *β* | *SE* | *p* | *Boot LLCI* | *Boot ULCI* |
| --- | --- | --- | --- | --- | --- | --- | --- |
| **Indirect effects** | |  |  |  |  |  |  |
| Age → | loneliness self-stigma | → Loneliness frequency | -0.149 | 0.030 | < 0.001 | -0.208 | -0.091 |
|  |  | → Loneliness intensity | -0.155 | 0.031 | < 0.001 | -0.216 | -0.095 |
|  |  | → Loneliness duration | -0.146 | 0.030 | < 0.001 | -0.205 | -0.087 |
| Total indirect effect of Loneliness self-stigma | | | -0.450 | 0.089 | < 0.001 | -0.624 | -0.277 |
| Age → | Meaning of loneliness | → Loneliness frequency | -0.068 | 0.018 | < 0.001 | -0.103 | -0.034 |
|  |  | → Loneliness intensity | -0.075 | 0.019 | < 0.001 | -0.113 | -0.037 |
|  |  | → Loneliness duration | -0.063 | 0.017 | < 0.001 | -0.096 | -0.030 |
| Total indirect effect of Meaning of loneliness | | | -0.206 | 0.052 | < 0.001 | -0.309 | -0.104 |
| **Total effects** | |  |  |  |  |  |  |
|  |  |  | -0.568 | 0.225 | 0.010 | -1.008 | -0.127 |

*Note.* Adjusting for gender, SES, living alone, and solitude.

**(4) the evaluation of the competing sequential (chain) mediation models**

Two competing sequential (chain) mediation models were conducted: Model 1 (age → meaning of loneliness → loneliness self-stigma → loneliness frequency, intensity, duration) and Model 2 (age → loneliness self-stigma → meaning of loneliness → loneliness frequency, intensity, duration).

The sequential indirect effects were found to be statistically significant (*ps* < 0.05). However, given the large sample size (*n* = 6875), these *p*-values should be interpreted with caution. The standardized sequential (chain) mediating effect sizes were examined instead of purely significance (Preacher & Kelley, 2011), which were exceptionally small (*βs* range from 0.005 to 0.015, each sequential mediating effect accounting for less than 4% of the total effect), and *R^2^* for total loneliness is 0.40 (for loneliness frequency, intensity, and duration are 0.136, 0.152, and 0.112 separately). In the context of psychological research on loneliness, these magnitudes are considered practically negligible. According to methodological references (Fan & Konold, 2010; Wu & Wen, 2011), the sequential mediating effects of the current sample explain virtually no meaningful variance in the outcome variable.

Furthermore, compared with the parallel mediation model, the competing sequential (chain) mediation model failed to meet the generally accepted cutoff criteria for adequate model fit (Hu & Bentler, 1999). Specifically, the sequential (chain) mediation models yielded RMSEA = 0.09 (> 0.80), whereas the proposed parallel model demonstrated better fit to the data (RMSEA = 0.01 (< 0.8)).

Table S16. *The model fit and mediating effects of competing sequential (chain) mediation models.*

| **Model** | | **Fit index and chain mediating effects** | | | | |
| --- | --- | --- | --- | --- | --- | --- |
| **Chain mediating model 1** | | *χ2/df* | *CFI* | *TLI* | *SRMR* | *RMSEA* |
|  | | 58.624 | 0.98 | 0.94 | 0.04 | 0.09 |
|  | | *β* | *SE* | *p* | *Boot LLCI* | *Boot ULCI* |
| Age → Meaning of loneliness → Loneliness self-stigma | → Loneliness frequency | -0.015 | 0.002 | < .001 | -0.018 | -0.012 |
|  | → Loneliness intensity | -0.015 | 0.003 | < .001 | -0.018 | -0.012 |
|  | → Loneliness duration | -0.014 | 0.001 | < .001 | -0.016 | -0.011 |
| **Chain mediating model 2** | | *χ2/df* | *CFI* | *TLI* | *SRMR* | *RMSEA* |
|  | | 58.261 | 0.98 | 0.94 | 0.04 | 0.09 |
|  |  | *β* | *SE* | *p* | *Boot LLCI* | *Boot ULCI* |
| Age → Loneliness self-stigma → Meaning of loneliness | → Loneliness frequency | -0.005 | 0.001 | < .001 | -0.004 | -0.006 |
|  | → Loneliness intensity | -0.006 | 0.001 | < .001 | -0.004 | -0.007 |
|  | → Loneliness duration | -0.005 | 0.001 | < .001 | -0.006 | -0.003 |

*Note.* Adjusting for gender, SES, living alone, and solitude.

# **Supplementary 5: LDA**

Table S17.

*The LDA model index of topic number in the younger age group (<= 39.2 years old).*

| *k* | Perplexity | Topic Coherence | NPMI |
| --- | --- | --- | --- |
| 1 | 86.04 | 0.39 | -0.02 |
| 2 | 89.78 | 0.44 | -0.03 |
| 3 | 92.28 | 0.40 | -0.05 |
| 4 | 95.88 | 0.40 | -0.03 |
| 5 | 98.72 | 0.41 | -0.04 |
| 6 | 101.502 | 0.41 | -0.04 |

*Note. k* refers to the number of topics, and NPMI refers to Normalized Pointwise Mutual Information.

Table S18.

*The LDA model index of topic number in the older age group (> 39.2 years old).*

| *k* | Perplexity | Topic Coherence | NPMI |
| --- | --- | --- | --- |
| 1 | 120.41 | 0.39 | -0.01 |
| 2 | 123.11 | 0.49 | -0.02 |
| 3 | 129.42 | 0.45 | -0.03 |
| 4 | 134.66 | 0.46 | -0.02 |
| 5 | 140.14 | 0.41 | -0.03 |
| 6 | 143.72 | 0.41 | -0.07 |

*Note. k* refers to the number of topics, and NPMI refers to Normalized Pointwise Mutual Information.


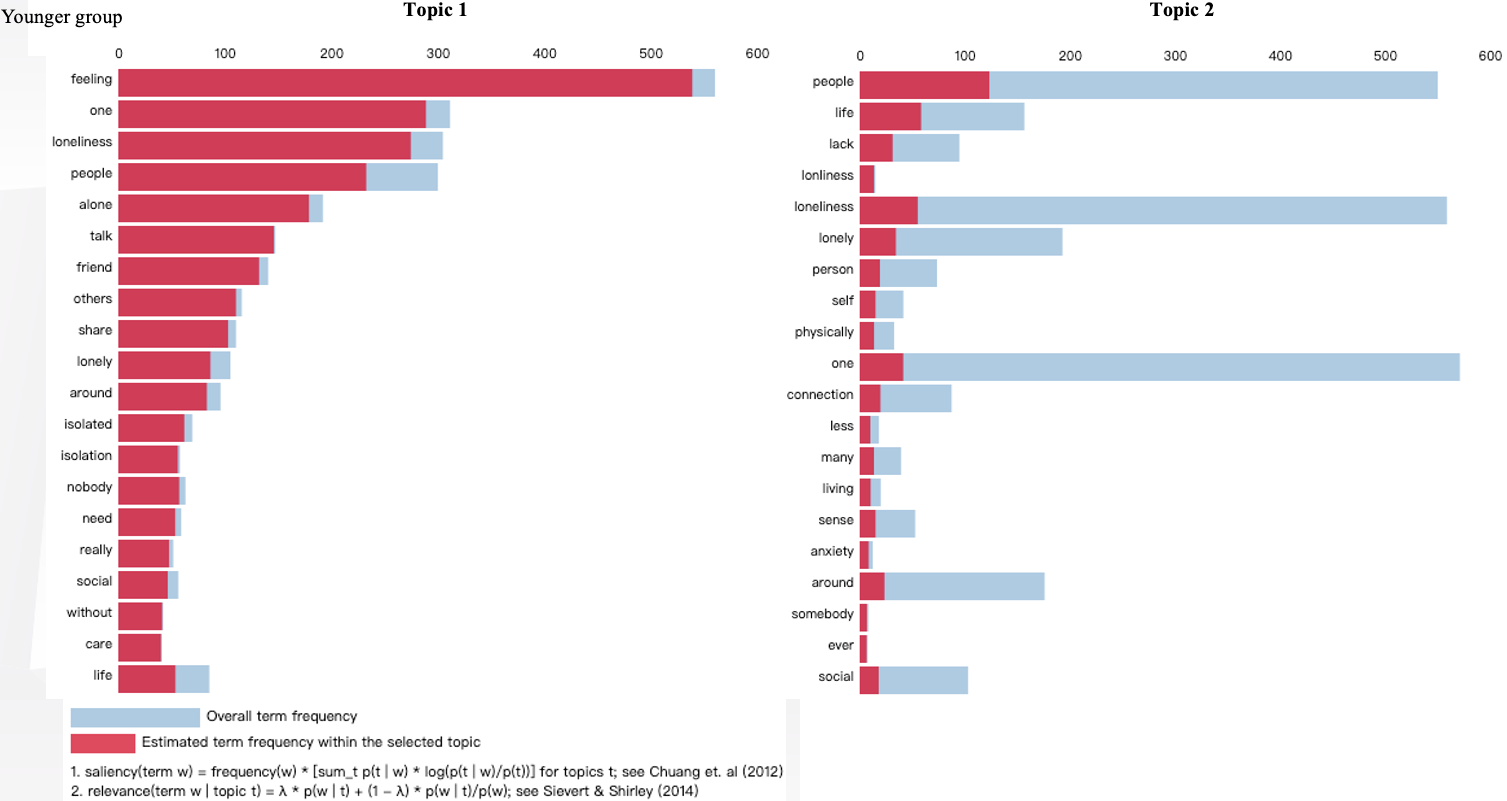


Figure S1. *Top-20 Most relevant words(terms) for each topic of the younger age group (<= 39.2 years old)*

*
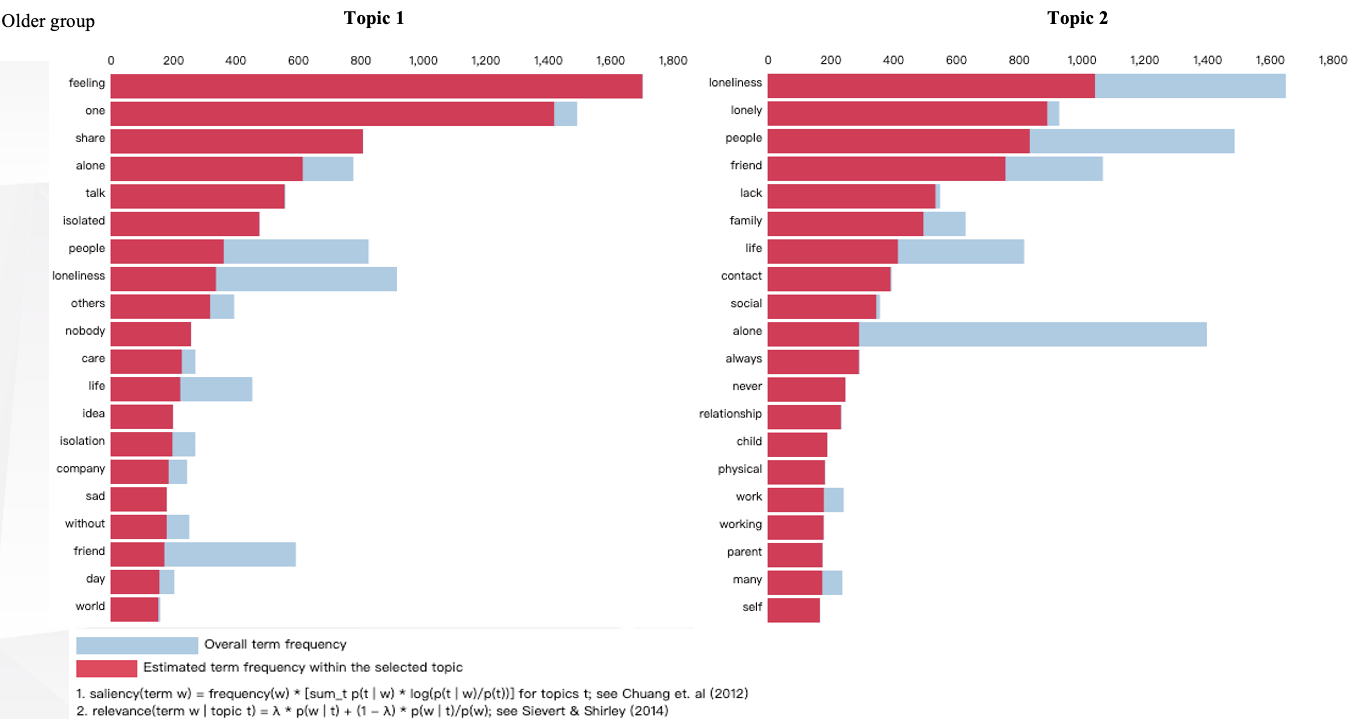
*

Figure S2. *Top-20 Most relevant words(terms) for each topic of the older age group (> 39.2 years old)*

Table S19. *The words and their distribution probabilities for each topic.*

| Topic 1 Limited Social Functions | | | | |  | Topic 2 Unsatisfactory Social Relationships | | | | |
| --- | --- | --- | --- | --- | --- | --- | --- | --- | --- | --- |
| younger age group (79.90%) | |  | older age group  (53.30%) | |  | younger age group (20.10%) | |  | older age group  (46.70%) | |
| words | probability |  | words | probability |  | words | probability |  | words | probability |
| feeling | 0.0700 |  | feeling | 0.0693 |  | people | 0.0348 |  | loneliness | 0.0269 |
| one | 0.0375 |  | one | 0.0578 |  | life | 0.0165 |  | lonely | 0.0230 |
| loneliness | 0.0357 |  | share | 0.0329 |  | loneliness | 0.0156 |  | people | 0.0216 |
| people | 0.0302 |  | alone | 0.0250 |  | one | 0.0117 |  | friend | 0.0196 |
| alone | 0.0233 |  | talk | 0.0227 |  | feeling | 0.0110 |  | lack | 0.0138 |
| talk | 0.0190 |  | isolated | 0.0194 |  | lonely | 0.0097 |  | family | 0.0128 |
| friend | 0.0172 |  | people | 0.0148 |  | lack | 0.0089 |  | life | 0.0107 |
| others | 0.0143 |  | loneliness | 0.0137 |  | alone | 0.0068 |  | contact | 0.0101 |
| share | 0.0134 |  | others | 0.0130 |  | around | 0.0066 |  | social | 0.0089 |
| lonely | 0.0112 |  | nobody | 0.0105 |  | connection | 0.0056 |  | alone | 0.0075 |
| around | 0.0108 |  | care | 0.0093 |  | person | 0.0054 |  | always | 0.0075 |
| isolated | 0.0080 |  | life | 0.0091 |  | social | 0.0051 |  | never | 0.0064 |
| nobody | 0.0074 |  | idea | 0.0081 |  | friend | 0.0044 |  | relationship | 0.0060 |
| isolation | 0.0073 |  | isolation | 0.0081 |  | support | 0.0043 |  | child | 0.0049 |
| life | 0.0070 |  | company | 0.0076 |  | sense | 0.0043 |  | physical | 0.0047 |
| need | 0.0069 |  | sad | 0.0073 |  | self | 0.0043 |  | work | 0.0046 |
| really | 0.0062 |  | without | 0.0073 |  | isolated | 0.0039 |  | working | 0.0046 |
| social | 0.0060 |  | friend | 0.0070 |  | physically | 0.0039 |  | parent | 0.0045 |
| without | 0.0053 |  | day | 0.0064 |  | very | 0.0039 |  | many | 0.0045 |
| care | 0.0052 |  | world | 0.0062 |  | many | 0.0038 |  | self | 0.0043 |
| company | 0.0052 |  | need | 0.0060 |  | share | 0.0038 |  | left | 0.0040 |
| family | 0.0051 |  | unable | 0.0058 |  | understood | 0.0037 |  | sometimes | 0.0040 |
| surrounded | 0.0048 |  | understands | 0.0057 |  | day | 0.0035 |  | long | 0.0039 |
| understand | 0.0048 |  | conversation | 0.0053 |  | contact | 0.0031 |  | single | 0.0039 |
| connection | 0.0048 |  | empty | 0.0052 |  | surrounded | 0.0031 |  | person | 0.0039 |

**References:**

Arnett, J. J. (2000). Emerging adulthood: A theory of development from the late teens through the twenties.*American Psychologist, 55*(5), 469-480. <https://doi.org/10.1037/0003-066X.55.5.469>

Chen, F. F. (2007). Sensitivity of goodness of fit indexes to lack of measurement invariance. *Structural Equation Modeling, 14*(3), 464–504. https://doi.org/10.1080/10705510701301834

Cheung, G. W., & Rensvold, R. B. (2002). Evaluating goodness-of-fit indexes for testing measurement invariance. *Structural Equation Modeling*, *9*(2), 233-255. <https://doi.org/10.1207/S15328007SEM0902_5>

Chuang, J., Manning, C. D., & Heer, J. (2012, May). Termite: Visualization techniques for assessing textual topic models. In *Proceedings of the International Working Conference on Advanced Visual Interfaces* (pp. 74-77).

Fan, X., & Konold, T. R. (2010). Statistical significance versus effect size. In P. Peterson, E. Baker, and B. McGaw (Eds.), *International encyclopedia of education* (3rd ed., Vol. 7, pp. 444–450). Oxford: Elsevier.

Hofstede, G. (1997). *Cultures and organizations: Software of the mind.* USA: McGraw-Hill.

Hu, L. T., & Bentler, P. M. (1999). Cutoff criteria for fit indexes in covariance structure analysis: Conventional criteria versus new alternatives. *Structural Equation Modeling: A Multidisciplinary Journal*, 6(1), 1–55. <https://doi.org/10.1080/10705519909540118>

Morbey, R. A., Todkill, D., Hughes, H. E., Charlett, A., & Elliot, A. J. (2025). Evaluation of age groupings used for syndromic surveillance. *medRxiv*, 2025-01. <https://doi.org/10.1101/2025.01.10.25320339>

Preacher, K. J., & Kelley, K. (2011). Effect size measures for mediation models: Quantitative strategies for communicating indirect effects. *Psychological Methods, 16*(2), 93–115. [https://doi.org/10.1037/a0022658](https://psycnet.apa.org/doi/10.1037/a0022658)

Reise, S. P., Scheines, R., Widaman, K. F., & Haviland, M. G. (2013). Multidimensionality and structural coefficient bias in structural equation modeling: A bifactor perspective. *Educational and Psychological Measurement*, *73*(1), 5-26. https://doi.org/10.1177/0013164412449831

Rodriguez, A., Reise, S. P., & Haviland, M. G. (2016a). Applying bifactor statistical indices in the evaluation of psychological measures. *Journal of Personality Assessment*, *98*(3), 223-237. https://doi.org/10.1080/00223891.2015.1089249

Rodriguez, A., Reise, S. P., & Haviland, M. G. (2016b). Evaluating bifactor models: Calculating and interpreting statistical indices. *Psychological Methods, 21*(2), 137–150. [https://doi.org/10.1037/met0000045](https://psycnet.apa.org/doi/10.1037/met0000045)

Russell, D., Peplau, L. A., & Cutrona, C. E. (1980). The revised UCLA Loneliness Scale: Concurrent and discriminant validity evidence. *Journal of Personality and Social Psychology, 39*(3), 472–480. [https://doi.org/10.1037/0022-3514.39.3.472](https://psycnet.apa.org/doi/10.1037/0022-3514.39.3.472)

Satorra, A., & Bentler, P. M. (2001). A scaled difference chi-square test statistic for moment structure analysis. *Psychometrika, 66*(4), 507–514. <https://doi.org/10.1007/BF02296192>

Sievert, C., & Shirley, K. (2014, June). LDAvis: A method for visualizing and interpreting topics. In *Proceedings of the International Working Conference on Advanced Visual Interfaces* (pp. 63-70).

Vincenzi, H., & Grabosky, F. (1987). Measuring the emotional/social aspects of loneliness and isolation. *Journal of Social Behavior and Personality*, *2*(2), 257-270.

Wu, Y., & Wen, Z. (2011). The statistical analysis procedure involving null hypothesis significance testing. *Psychological Science, 34*, 230–234.
